# Novel polyhydroxyalkanoate–graphene oxide composites with potential for clinical application against bacterial implant-associated infections in septic surgery

**DOI:** 10.3389/fbioe.2025.1671682

**Published:** 2025-09-25

**Authors:** A. Paxinou, R. Nigmatullin, G. Paterakis, L. Sygellou, R. Viebahn, C. Galiotis, J. Salber, I. Roy

**Affiliations:** ^1^ Faculty of Science and Technology, College of Liberal Arts, University of Westminster, London, United Kingdom; ^2^ Foundation of Research and Technology Hellas, Institute of Chemical Engineering and High Temperature Chemical Processes (FORTH/ICE-HT), Patras, Greece; ^3^ Department of Surgery, Knappschaft Kliniken Universitätsklinikum Bochum, Ruhr University Bochum, Bochum, Germany; ^4^ Experimental Surgery, Ruhr University Bochum, Bochum, Germany; ^5^ School of Chemical, Materials and Biological Engineering, Faculty of Engineering, University of Sheffield, Sheffield, United Kingdom; ^6^ Insigneo Research Institute, University of Sheffield, Sheffield, United Kingdom

**Keywords:** polyhydroxyalkanoates, graphene oxide, composites, antibacterial, neuronal

## Abstract

**Introduction:**

Implant-associated infections are a major clinical challenge, often leading to implant failure, revision surgeries, and increased healthcare costs. The development of advanced biomaterials with inherent antimicrobial properties is critical to address this issue. In this study, we present novel two-dimensional (2D) composite films based on polyhydroxyalkanoates (PHAs) combined with graphene oxide (GO) to confer both antimicrobial activity and tailored mechanical properties.

**Methods:**

Composites with varying GO concentrations (0.5, 2, and 5 wt%) were fabricated using the solvent casting method, using both a short-chain-length PHA, P(3HB) and a medium-chain-length PHA, P(3HO-co-3HD). Physicochemical characterization (scanning electron microscopy (SEM), X-ray photoelectron spectroscopy (XPS), Raman spectroscopy, X-ray diffraction (XRD), differential scanning calorimetry (DSC), and mechanical testing) confirmed successful GO incorporation, changes in surface morphology, and modifications in thermal and mechanical properties.

**Results:**

Notably, the incorporation of 2 wt% GO into P(3HB) increased the Young’s modulus from 776 ± 15 MPa to 1,055 ± 28 MPa, indicating enhanced stiffness. Antibacterial testing using ISO 22196 against Staphylococcus aureus and Escherichia coli revealed that P(3HB)/2 wt% GO exhibited the highest antibacterial efficacy. In contrast, the 5 wt% GO composite showed reduced antibacterial activity, likely due to GO agglomeration. Moreover, in vitro cytocompatibility assays using L929 fibroblasts and NG108-15 neuronal cells demonstrated high cell viability across all composites, indicating high cytocompatibility.

**Discussion/Conclusion:**

These findings highlight the potential of PHA/GO composites as sustainable, antimicrobial biomaterials for future use in implantable devices for septic surgical applications.

## 1 Introduction

In modern medicine, biofilm-associated implant-related infections pose a major problem. Bacteria adhere to implant surfaces and evade the immune response and antibiotic therapy by forming biofilms (Kuilla et al., 2010; Zou et al., 2016). The frequent and widespread antibiotic resistance of certain microbes limits the effectiveness of conventional treatments (Mohammed et al., 2020; Er et al., 2022). Bacterial colonization and biofilm formation represent a major challenge in chronic disease management and surgical interventions, contributing significantly to morbidity in conditions such as chronic obstructive pulmonary disease (COPD) and increasing susceptibility to opportunistic infections (Li et al., 2025). Despite extensive research, conventional biomaterials often lack antibacterial properties, increasing the risk of implant failure and revision surgeries (Perreault et al., 2013; Kumar et al., 2019). Although peri-operative or intra-operative bacterial contamination of implants is possible, bacterial colonization via bodily fluids is a more common cause of infection, leading to biofilm formation on medical devices such as catheters and joint prostheses (Wang et al., 2022; Qin et al., 2020). This contributes to high revision surgery rates, affecting pacemakers, heart valves, and stents (Perreault et al., 2013; de Moraes et al., 2015). Implant removal is often necessary, even if only one of multiple implants is affected, which not only increases healthcare expenses and prolongs patient recovery time but also has significant socio-economic consequences (Mohammed et al., 2020; Malisz and Świeczko-Żurek, 2023). To address this, research focuses on developing antimicrobial biomaterials to prevent bacterial adhesion and reduce infection risks (Er et al., 2022; Sundar et al., 2014). An alternative to conventional antibiotics involves searching for different compounds that can provide antibacterial efficacy via different or multiple mechanisms of action.

Graphene-based materials (GBMs) have garnered significant attention due to their unique physicochemical properties, particularly in nanoscience and biomedicine (Kuilla et al., 2010). Their antibacterial and biocompatible properties make them promising for biosensors, tissue engineering, drug delivery, and cancer therapy. Among these, graphene oxide (GO) stands out due to its high surface area and oxygen functional groups, which enhance dispersibility and bioactivity (Bellier et al., 2022; Malisz and Świeczko-Żurek, 2023; Arabinda et al., 2024).

GO exhibits potent antibacterial activities, particularly for *Staphylococcus aureus* and *Escherichia coli*, through mechanisms such as membrane disruption, oxidative stress, and bacterial entrapment (Zou et al., 2016; Rojas-Andrade et al., 2017; Kumar et al., 2019). Studies have confirmed GO’s superior antibacterial efficiency compared to other GBMs, with its effectiveness attributed to several factors, such as sheet size, concentration, and dispersion (Liu et al., 2011; 2012; Hu et al., 2010). GO–polyamide and GO–Ag composites further enhance antibacterial activity against multi-drug-resistant strains such as MRSA (Perreault et al., 2013; de Moraes et al., 2015). More recently, GO has also been shown to confer both bactericidal and osteogenic properties when applied to biomedical substrates. For example, a self-assembling GO coating on polyetheretherketone (PEEK) improved antibacterial efficacy while simultaneously promoting osteoblast differentiation and proliferation (Huang et al., 2024). This dual functionality underscores the potential of GO-modified polymer surfaces to prevent bacterial colonization while supporting tissue integration, a combination particularly relevant for long-term implant performance.

Beyond antimicrobial applications, GBMs support osteogenic differentiation, stem cell adhesion, and nerve regeneration (Hoda et al., 2015; Vera-Sanchez et al., 2016; Henriques et al., 2020). Their biocompatibility has been validated *in vitro* and *in vivo*, with minimal toxicity in various models (Pinto et al., 2013; Mohan et al., 2018; Williams et al., 2023). GBM-based scaffolds show potential for spinal cord repair and peripheral nerve regeneration, with studies suggesting that their effectiveness may rival that of autologous grafts (Wang et al., 2022; Qian et al., 2021). With their versatility and promising biomedical applications, GBMs continue to be explored as next-generation materials for antibacterial treatments, tissue engineering, and neural repair (Hoda et al., 2015; Vera-Sanchez et al., 2016; Henriques et al., 2020).

Polyhydroxyalkanoates (PHAs) have been used as matrices in biomedical applications for several years. These are promising biopolymers for biomedical composites due to their favorable physicochemical and biological properties (Rai et al., 2008; Możejko-Ciesielska and Kiewisz, 2016). They are produced naturally by bacterial fermentation utilizing renewable resources, offering a sustainable alternative to petroleum-based plastics. They are classified into short-chain-length PHAs (scl-PHAs) and medium-chain-length PHAs (mcl-PHAs), with scl-PHAs being stiff and brittle (melting temperature: 160 °C–180 °C, glass transition ∼0 °C), while mcl-PHAs are more flexible, with lower melting (40 °C–60 °C) and glass transition temperatures (−50 °C to 25 °C) (Możejko-Ciesielska and Kiewisz, 2016; Puppi et al., 2019; Basnett et al., 2018). These biopolymers have demonstrated significant potential in drug delivery, bone and nerve tissue engineering, and wound healing. For example, poly(3-hydroxybutyrate) (P(3HB)) microspheres have been used to encapsulate rifampicin and tetracycline, ensuring prolonged release with minimal side effects (Kassab et al., 1999; Sendil et al., 1999). P(3HB) composites reinforced with hydroxyapatite promoted rapid bone formation and integration (Doyle et al., 1991; Luklinska and Bonfield, 1997), while antibiotic-free antibacterial scaffolds incorporating thioester groups or selenium- and strontium-doped hydroxyapatite have shown promise for bone tissue engineering (Marcello et al., 2021). Moreover, in soft tissue engineering, P(3HB)-based scaffolds supported keratinocyte attachment and proliferation, aiding wound healing (Rai et al., 2008). In nerve tissue engineering, P(3HB) was used for successfully bridging 2 mm nerve gaps in cats and 10 mm gaps in rats, showing results comparable to those of autografts (Misra et al., 2006; Chen, 2009; Rai et al., 2008; Masood, 2016). More recently, mcl-PHA blends, such as poly(3-hydroxyoctanoate-*co*-3-hydroxydecanoate) (P(3HO-*co*-3HD)), have been used in nerve conduits, supporting neuronal cell proliferation and promoting neurite growth due to their favorable mechanical properties (Lizarraga-Valderrama et al., 2015). Recent reviews further highlight that blending PHAs with other polymers or incorporating nanofillers can significantly modify their crystallization behavior, morphology, and performance (Eesaee et al., 2022). Such modifications can be tailored to improve stiffness, ductility, or barrier properties, thereby enhancing their suitability for implantable devices and antimicrobial materials.

Despite the increase in biomedical studies with GBMs, there are only a few reports on the correlations between GBM concentration and its effectiveness against bacterial strains without simultaneously negatively affecting eukaryotic cells.

This study investigates the physicochemical and biological aspects of the developed two-dimensional (2D) composites, based on the combination of PHAs with GO, evaluating their *in vitro* biocompatibility and antibacterial activity with respect to various eukaryotic and prokaryotic cells. The present study explores the feasibility of developing biomedical devices containing effective antibacterial agents that can combat widespread bacterial infections while exhibiting no toxicity toward mammalian cells.

## 2 Materials and methods

### 2.1 Materials and chemicals

Chemicals, reagents, and materials for the production and characterization of PHAs and its composites were purchased from Sigma-Aldrich Company Ltd. (United Kingdom and Germany), VWR International (United Kingdom), Thermo Fisher Scientific (United Kingdom and Germany), and FORTH/ICE-HT Institute, Patras (Greece).

### 2.2 Synthesis of graphene oxide-antibacterial compound

**FIGURE 1:**
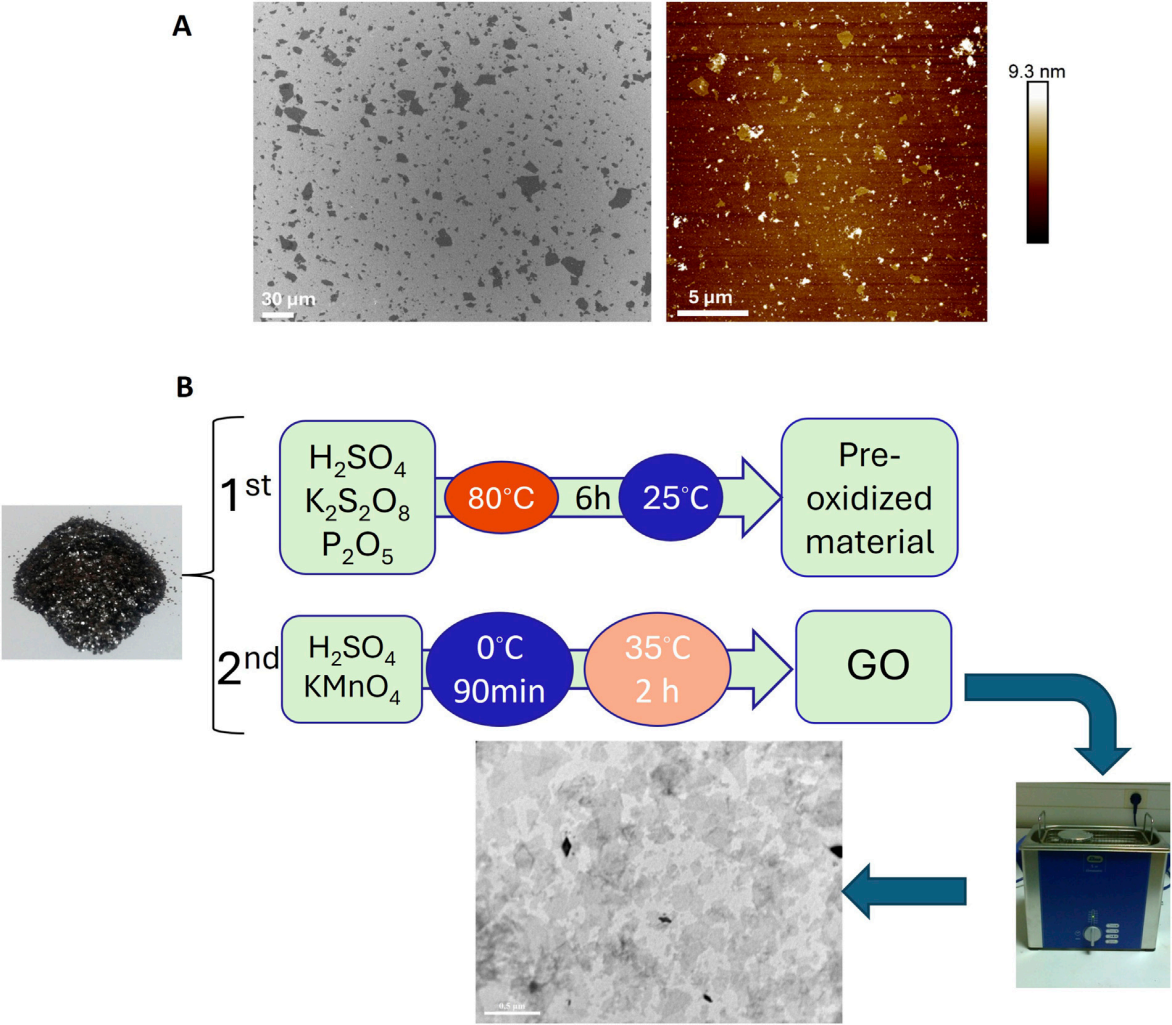
**(A)** SEM images of GO material and **(B)** schematic representation of the two-step oxidation process used for graphene oxide (GO) synthesis.

GO was synthesized from natural graphite flakes (NGS Naturgraphit GmbH, Germany) using a modified Hummers method (Hummers and Offeman, 1958), as previously reported (Paterakis et al., 2021). The exact synthetic protocol used was as follows: 10 g of natural graphite flakes were added to a solution of 6.5 g potassium persulfate (K_2_S_2_O_8_) and 8.8 g of phosphorus pentoxide (P_2_O_5_) in 37.5 mL concentrated sulfuric acid (H_2_SO_4_, 96%). The mixture was then placed in an oil bath and heated at 80 ^°^C for 1 h with constant stirring. After 1 h, the mixture was stirred and allowed to cool to room temperature for 5 h. Deionized water was carefully added to the mixture, which was then filtered and washed using a PTFE membrane filter with a 0.45 μm pore size (Sigma-Aldrich) until the pH was neutralized (7.0). Finally, the pre-oxidized graphite powder was air-dried at ambient conditions overnight. Subsequently, the powder was added to an ice-cold flask containing 230 mL of H_2_SO_4_ (96%), with constant stirring, and 32 g of potassium permanganate (KMnO_4_) was added. The ice bath was removed, and the mixture was stirred at 35 ^°^C for 2 h (Figure 1B). The reaction was quenched by careful addition of 2 L of deionized water and 25 mL of hydrogen peroxide (H_2_O_2_, 30%). The mixture was then filtered and washed with a 1:10 aqueous HCl solution. The solid material was dispersed in deionized water and dialyzed, using a flat cellulose membrane dialysis tubing with a width of 25 mm (Sigma-Aldrich), until the pH was 7.0. Finally, monolayers of the GO material were collected by a combination of sonication and centrifugation with an average lateral size below 1 μm, as shown in the scanning electron microscopy (SEM) image in Figure 1A.

### 2.3 Bacterial strains and culture conditions

*Bacillus subtilis* OK2 and *Pseudomonas mendocina* CH50 were obtained from the University of Westminster culture collection. The strains were cultured at 30 °C for 16 h in a shaking incubator (200 rpm).

The antibacterial studies were carried out using Gram-positive *Staphylococcus aureus* ATCC^®^ 29213™ and Gram-negative *Escherichia coli* ATCC^®^ 25922™. The bacterial culture was revived either from glycerol stock at −20 °C or glycerol stock at −80 °C. The strains were recovered by culturing them on blood agar plates at 37 °C overnight for 16 h. A single colony from the grown bacteria on the blood agar plates was collected with a sterile loop and re-suspended in a tube containing 2 mL of sterile, isotonic, aqueous saline (NaCl) solution (0.9%, w/v). A densitometer was used to obtain a bacterial suspension of 0.5 McFarland, which corresponds to a turbidity equivalent to approximately 1.5 × 10^8^ CFU/mL.

#### 2.3.1 Production of polyhydroxyalkanoates from *Bacillus subtilis OK2* and *Pseudomonas mendocina CH50*

The linear polyesters were produced using glycose as the sole carbon source under nitrogen-limiting conditions. For the fabrication of the P(3HB), *B. subtilis OK2* was used as the producer microorganism, using a glucose concentration of 35 g/L, whereas for the copolymer of P(3HO-*co*-3HD), *P. mendocina* CH50 with a glucose concentration of 20 g/L was used as described by Basnett et al. (2018) and Lukasiewicz et al. (2018) The fermentation process for obtaining the different biopolymers was carried out in batch-mode fermenters at the Department of Life Sciences, University of Westminster, London, United Kingdom.

### 2.4 Preparation of composite film samples

Polymer solutions of neat P(3HB) and P(3HO-*co*-3HD) were prepared by dissolving them in chloroform (5% w/v) at room temperature and stirring for 24 h. For the PHA/GO composites, the desired amount of the filler (0.5, 2, and 5 wt%) was simultaneously dispersed into the same solvent (chloroform) and sonicated for 30 min. Both solutions were then mixed and stirred constantly for 24 h. Prior to solvent casting, the solution containing the polymer and the filler was sonicated for a further 45 min to ensure the optimal dispersion of GO.

### 2.5 Physicochemical characterization of films

#### 2.5.1 Scanning electron microscopy

The surface topography of the samples was studied via scanning electron microscopy (SEM) (Philips XL30 Scanning Electron Microscope). Samples were mounted on aluminum stubs, coated with gold using an EMITECH-K550 gold-sputtering device for 2 min, and then examined under a scanning electron microscope. The scanning electron microscope was operated at an acceleration voltage of 10 kV with a working distance of 10 cm. The analysis was carried out at the Department of Biomaterials and Tissue Engineering, Eastman Dental Institute, University College London, United Kingdom.

#### 2.5.2 Water contact angle measurements

Static water contact angle (WCA) studies were carried out to investigate the wettability of the PHA films and their GO composites. The studies were carried out using a Theta Lite Optical Tensiometer (Biolin Scientific, Manchester, United Kingdom) operated with OneAttension software. An amount of 200 μL of deionized water was dropped on the surface of the films using a gas-tight micro-syringe. The contact angle of the droplet on the specimen surface was recorded from 10 images captured at various surface points, with a frame interval of 1 s. All experiments were carried out in triplicate, and the mean value was calculated.

#### 2.5.3 Raman spectroscopy

A MicroRaman setup (inVia Reflex, Renishaw, United Kingdom) equipped with an Olympus MPLN100x objective (NA = 0.90) was used to focus the beam on the samples at a wavelength of 785 nm. The laser power was kept below 1.5 mW on the sample to avoid laser-induced local heating. Raman measurements were carried out at the Foundation for Research and Technology, Hellas/Institute of Chemical Engineering Sciences, Patra, Greece, and data analysis was carried out at the University of Westminster.

#### 2.5.4 X-ray diffraction

X-ray diffraction (XRD) diffractograms were obtained from a Bruker D8 Advance Diffractometer in flat plate geometry, equipped with Cu K_a_ radiation and a Ni filter. Data were collected from 5° to 100° (angle) with a primary beam slit size of 0.6 mm. The XRD measurements were carried out at the Foundation for Research and Technology, Hellas/Institute of Chemical Engineering Sciences, Patra, Greece, and data analysis was carried out at the University of Westminster.

#### 2.5.5 X-ray photoelectron spectroscopy

The photoemission experiments were carried out in an ultra-high vacuum (UHV) system equipped with a dual anode Mg/Al X-Ray gun. The unmonochromatized AlKα line at 1,486.6 eV and an analyzer pass energy of 97 eV were utilized, which resulted in a full width at half maximum (FWHM) of 1.7 eV for the Ag3d_5/2_ peak. The X-ray photoelectron spectroscopy (XPS) core-level spectra were analyzed using a fitting routine that decomposes each spectrum into individual mixed Gaussian–Lorentzian peaks following Shirley background subtraction. The samples were pressed into pellets. The analyzer’s kinetic energy scale was calibrated according to ASTM-E 902-88. Survey Scans and O 1s and C 1s core-level spectra were recorded in detail.

#### 2.5.6 Differential scanning calorimetry

The thermal properties of the samples were analyzed using a DSC 214 Polyma (Netzsch, Germany) equipped with an Intracooler IC 70 cooling system, enabling measurements from −70 °C. A sample mass of approximately 5 mg was exposed to cooling and heating ramps to allow manifestation of the glass transition and sample melting. The thermograms were used for the determination of the melting temperature (T_m_) and glass transition temperature (T_g_) and calculation of melting enthalpy. The encapsulated samples in aluminum crucibles were heated at a rate of 10 °C per min from −70 °C to 170 °C (mcl-PHAs) or 200 °C (scl-PHAs) and held at these temperatures for 2 min to remove the thermal history of the samples. The samples were then cooled to −70 °C at a rate of 20 °C per min and heated again at a rate of 10 °C per min. The thermograms of the second cycle were used for analysis using *Proteus* 7.0 software.

For the P(3HB) samples, the percentage of crystallinity (X_c_%) was calculated from the melting peak areas using the following equation:

$$X_{c\%}=\frac{\Delta H_{m}}{\Delta H^{0}}*100,$$

where ΔH_m_ is the melting enthalpy of the material/composite and ΔH^0^ is the melting enthalpy of fully crystallized P(3HB), which is 146.6 J/g for P(3HB) (Barham et al., 1984; Ho et al., 2014).

The enthalpy of fusion (ΔH_m_) of the composite samples was normalized (ΔH^n^_m_) to account for the weight fraction of the filler (w_f_) in the corresponding composites:

$$\Delta H_{m}^{n}=\frac{\Delta H_{m}}{\left(1-w_{f}\right)}.$$

#### 2.5.7 Mechanical analysis

Tensile testing was conducted using the Instron 5940 testing system. Polymer strips were cut out from films previously made by the solvent casting technique with a pre-defined size of 5 mm wide and 35 mm long. The thickness and width of each specimen were measured at several locations, and the average values were used to calculate the cross-sectional area. The samples were subjected to tensile testing until fracture. The deformation rate was set at 5 mm per minute for the scl and 10 mm per minute for the mcl samples, and the stress–strain curve was used to calculate the Young’s modulus (E), elongation at break (ε_b_), and ultimate tensile strength (UTS) following ASTM D882–10 (Lukasiewicz et al., 2018; Marcello et al., 2021).

### 2.6 Biological characterization of films

#### 2.6.1 Direct contact test—ISO 22196 (Japanese test method JISZ 2801)

The antibacterial properties of the composites were assessed using ISO 22196 (Japanese test method JISZ 2801) against *S. aureus* (ATCC^®^ 29213™) and *E. coli* (ATCC^®^ 25922™) using non-modified PHAs—P(3HB) (scl-PHA) and P(3HO-*co*-3HD) (mcl-PHA)—as control samples. The samples were tested in the form of discs, and all tests were performed in triplicate. UV sterilization was done for 15 min before the inoculation of the samples with 10 µL of the bacterial culture adjusted to a concentration of 3–5 × 10^5^ CFU/mL. The samples were placed into agar plates and incubated under static conditions at 37 °C for 24 h. Phosphate-buffered saline (PBS) was used to recover the viable bacterial cells and plated on agar plates using the drop plate technique. After 24 h of static incubation, the number of viable cells was determined through the colony-forming unit method, and antibacterial activity was expressed using the following formula:

$$R=\log\left(a\right)\;\left[control\right]–\;\log\left(a\right)\;\left[modified\right],$$

where a is the average of the viable bacterial cells recovered after 24 h expressed as CFU/(ml*cm^2^).

#### 2.6.2 *In vitro* direct cytotoxicity evaluation

The L929 murine fibroblasts were purchased from DSMZ (German Collection of Microorganisms and Cell Cultures GmbH), and NG108-15 neuronal cells (Cat. No. 88112302) were purchased from the European Collection of Authenticated Cell Cultures (ECACC). Cell culture materials were purchased from Sigma-Aldrich (Dusseldorf, Germany). Live/Dead Staining Kit II for mammalian cells (Invitrogen, Thermo Fisher Scientific, Paisley, United Kingdom) was purchased from PromoCell (Heidelberg, Germany). CytoTox-ONE^TM^ Homogeneous Membrane Integrity Assay and CellTiter-Blue^®^ Cell Viability Assay were purchased from Promega Corporation, Madison, United States.

L929 murine fibroblasts were grown in RPMI medium under a humidified atmosphere of 5% CO_2_ at 37 °C. The RPMI medium was supplemented with 10% (v/v) fetal bovine serum (FBS) and 1% (w/v) penicillin/streptomycin. Experiments were carried out only when confluency of 70%–80% was achieved. After trypsinization, 3 × 10^4^ cells were seeded per sample. The experiments were carried out in triplicate. The L929 cells were used between passages 2–9.

NG108-15 neuronal cells were grown in Dulbecco’s modified Eagle medium (DMEM) under a humidified atmosphere of 5% CO_2_ at 37 °C. The DMEM was supplemented with 10% (v/v) fetal calf serum (FCS), 1% (w/v) glutamine, 1% (w/v) penicillin/streptomycin, and 0.5% (w/v) amphotericin B. Cells were used in the experiments only after reaching 70%–80% confluence. Cells were trypsinized, and 3 × 10^4^ cells were seeded directly on the samples. All the cell culture experiments were performed at Bochum University in Germany.

The CellTiter-Blue Cell Viability Assay was used to evaluate the cytocompatibility of the samples. In brief, after the seeding period of the samples with the respective cell line, the supernatant was removed and 100 μL of CellTiter-Blue reagent was added to the samples and incubated for 2 h in a humidified atmosphere of 5% CO_2_ in air at 37 °C. Triton X-100, which caused cell lysis, was used as a negative control solution, while the tissue culture polystyrene (TCPS) and pristine polymers were considered positive controls. To quantify cell viability, after 2 h of incubation, aliquots of the reagent were transferred to a black 96-well plate, and fluorescence was measured at 560 nm. This experimental process was adapted from the International Organization for Standardization (ISO) norm 10993-5:2009 (International Organization for Standardization, 2009). Finally, the staining solution containing 1 µL/mL ethidium homodimer-III (red) and 0.25 µL/mL calcein AM (green) was added to the cells. The images were acquired using an inverted fluorescence microscope (Olympus IX51).

### 2.7 Statistical analysis

All the experiments were conducted in triplicate and reported as the mean value ± standard deviation. Statistical analysis was performed using GraphPad Prism 8 software, applying one-way analysis of variance (ANOVA) followed by Tukey’s post hoc test for comparisons between data groups. Differences were considered significant when *p*-values were lower than 0.05 (∗). Statistical significance determination is represented as *p ≤ 0.05, **p ≤ 0.01, and ***p ≤ 0.001.

## 3 Results

### 3.1 Physicochemical characterization of 2D antibacterial PHA/GO composites

**FIGURE 2:**
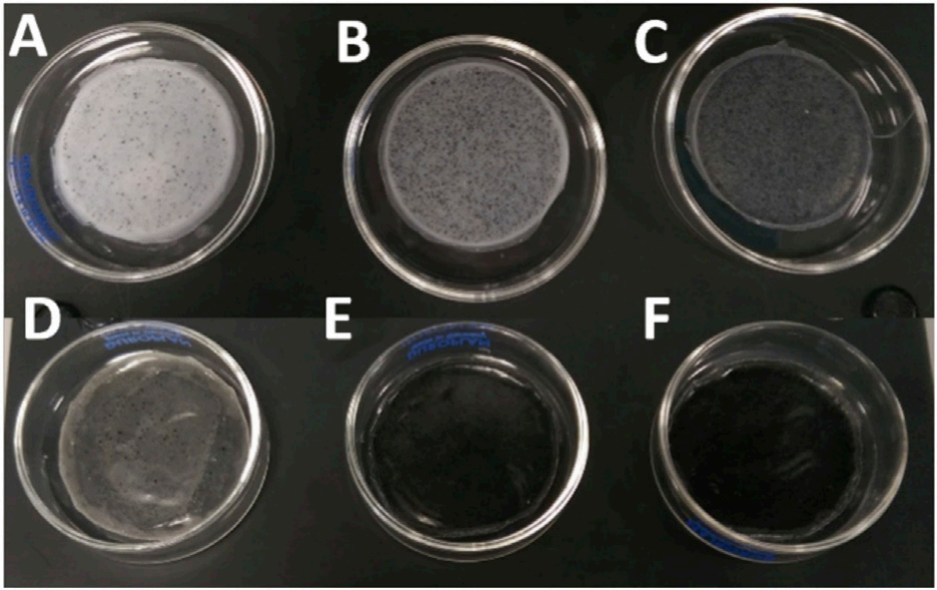
Composites of P(3HB) and P(3HO-*co*-3HD) with different weight concentrations of GO. **(A)** P(3HB)/0.5 wt% GO, **(B)** P(3HB)/2 wt% GO, **(C)** P(3HB)/5 wt% GO, **(D)** P(3HO-*co*-3HD)/0.5 wt% GO, **(E)** P (3HO-co-3HD)/2 wt% GO, and **(F)** P(3HO-*co*-3HD)/5 wt% GO.

The fabrication of the 2D antibacterial PHA/GO composite substrates was conducted via the solvent casting technique as described above. Samples for both scl P(3HB) and mcl P(3HO-*co*-3HD) polymer matrices were prepared with GO loadings of 0.5, 2, and 5 wt% with respect to polymer mass. The visual appearance of the fabricated composite films is shown in Figure 2. In the case of the homopolymer P(3HB), the composite film with 0.5 wt% GO appeared whitish with relatively homogeneously distributed, black speckles. With increasing mass fraction of GO from 2.0 wt% to 5.0 wt%, the amount of optically visible particles, presumably consisting of GO aggregates, increased, changing the optical impression from greyish to anthracite. In the case of the copolymer P(3HO-*co*-3HD), the composite film still appeared almost transparent after the addition of 0.5 wt% GO, while the composite films appeared deep black with 2.0 wt% GO and higher concentrations of GO (Figures 2D–F).

#### 3.1.1 Scanning electron microscopy of the PHA and PHA/GO composite films

**FIGURE 3:**
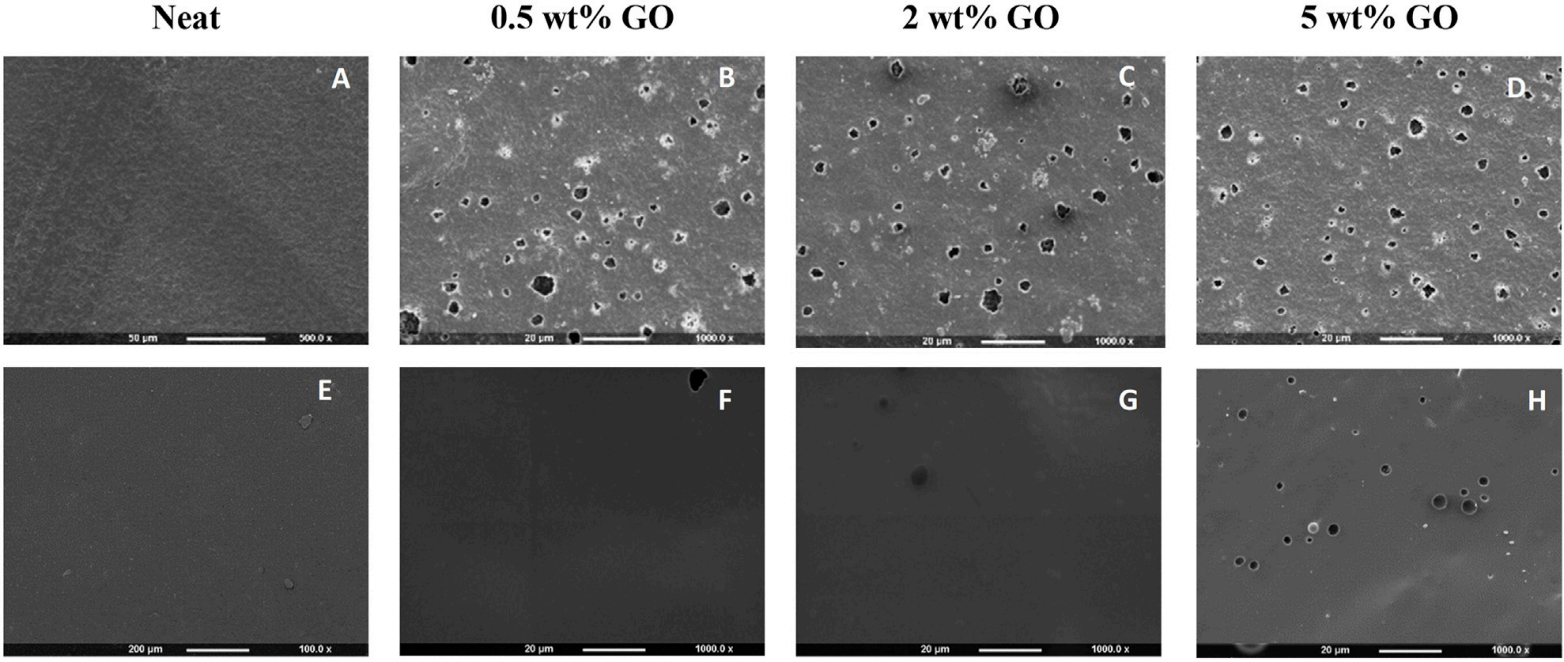
SEM images of **(A)** neat P(3HB), **(B–D)** P(3HB) composites containing 0.5, 2, and 5 wt% GO (from left to right), **(E)** neat P(3HO-*co*-3HD), and **(F–H)** P(3HO-*co*-3HD) composites containing 0.5, 2, and 5 wt% GO (from left to right).

SEM investigations were carried out to morphologically verify the GO nanoparticle aggregation during composite film preparation and their distribution in the two PHA polymer matrices P(3HB) and P(3HO-*co*-3HD). Figure 3 shows the effect of the addition of GO on the surface morphology of the composite films prepared using P(3HB) and P(3HO-*co*-3HD) as the polymer matrix, compared with the neat films. The neat P(3HB) film (Figure 3A) displayed a smooth and homogenous surface, which was noticeably altered by the addition of a low GO load (0.5 wt%). With the addition of the different loadings of GO into the P(3HB) matrix, the SEM micrographs revealed the presence of dispersed GO aggregates randomly distributed on the polymer surface. The images indicate that P(3HB) composites exhibit significant differences in surface morphology compared to the neat polymer, with randomly distributed irregular GO particles.

The SEM micrograph of neat P(3HO-*co*-3HD) showed an intact, smooth-surface without any apparent defects on the substrate. The lower concentration of GO (0.5 wt%) did not cause alterations on the polymer surface. With an increase in GO content, irregularly distributed GO particles were observed on the surface of the composites, as shown in Figures 3G,H.

#### 3.1.2 Surface wettability of the PHA and PHA/GO composite films

The surface wettability of the PHA films and their respective PHA/GO composites was evaluated using the static WCA method.

**FIGURE 4:**
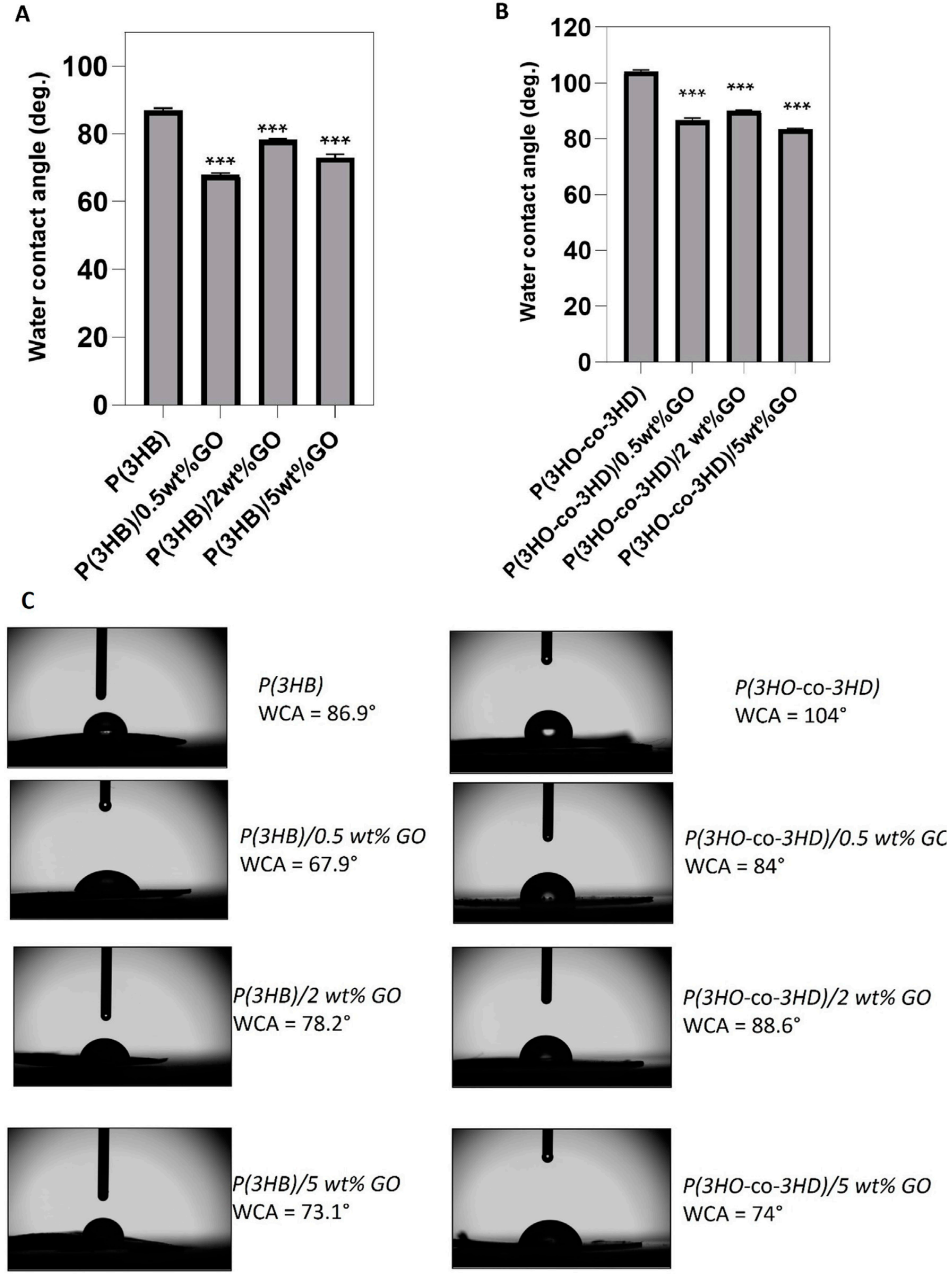
Water contact angle measurements for neat PHA films and their GO composites (n = 3). **(A)** WCA values of P(3HB) and its composites; **(B)** WCA values of P(3HO-co-3HD) and its composites; **(C)** side-view images of water droplets on film surfaces with labeled WCA values. Left: P(3HB) series; right: P(3HO-*co*-3HD) series. Statistical significance was determined for neat vs. composite films (*p ≤ 0.05, **p ≤ 0.01, ***p ≤ 0.001).

The addition of 0.5, 2, and 5 wt% GO resulted in WCA of 67.9° ± 0.4, 78.2° ± 0.4, and 73.1° ± 0.8, respectively (Figure 4A, C), while neat P(3HB) exhibited a WCA of 86.9° ± 0.6°. Statistical analyses showed that the differences in water contact angle between all the composite P(3HB)/GO films and the neat P(3HB) were significant (*p*-value < 0.05). Given that the WCA values of the composites were lower than 90° and lower than that of the neat homopolymer, P(3HB) composites are considered hydrophilic substrates.

The water contact angle of P(3HO-*co*-3HD) was 104°, where the addition of 0.5, 2, and 5 wt% GO resulted in a reduction of the water contact angle values of 17.3%, 13.5%, and 19.8%, respectively (Figure 4B). Statistical analysis of the P(3HO-*co*-3HD)/GO composites showed significant differences compared to the neat P(3HO-*co*-3HD) (***p ≤ 0.001).

#### 3.1.3 X-ray photoelectron spectroscopy analysis of the PHA and PHA/GO composite films

XPS measurements and analysis were performed to determine the relative atomic composition and the molecular bonding ratios between different atoms within a surface voxel with an angle-dependent penetration depth of approximately 10 nm. The surface functional groups and the electron transition states of carbon and oxygen in the neat PHAs and their composites with 5 wt% GO composites were analyzed.

**FIGURE 5:**
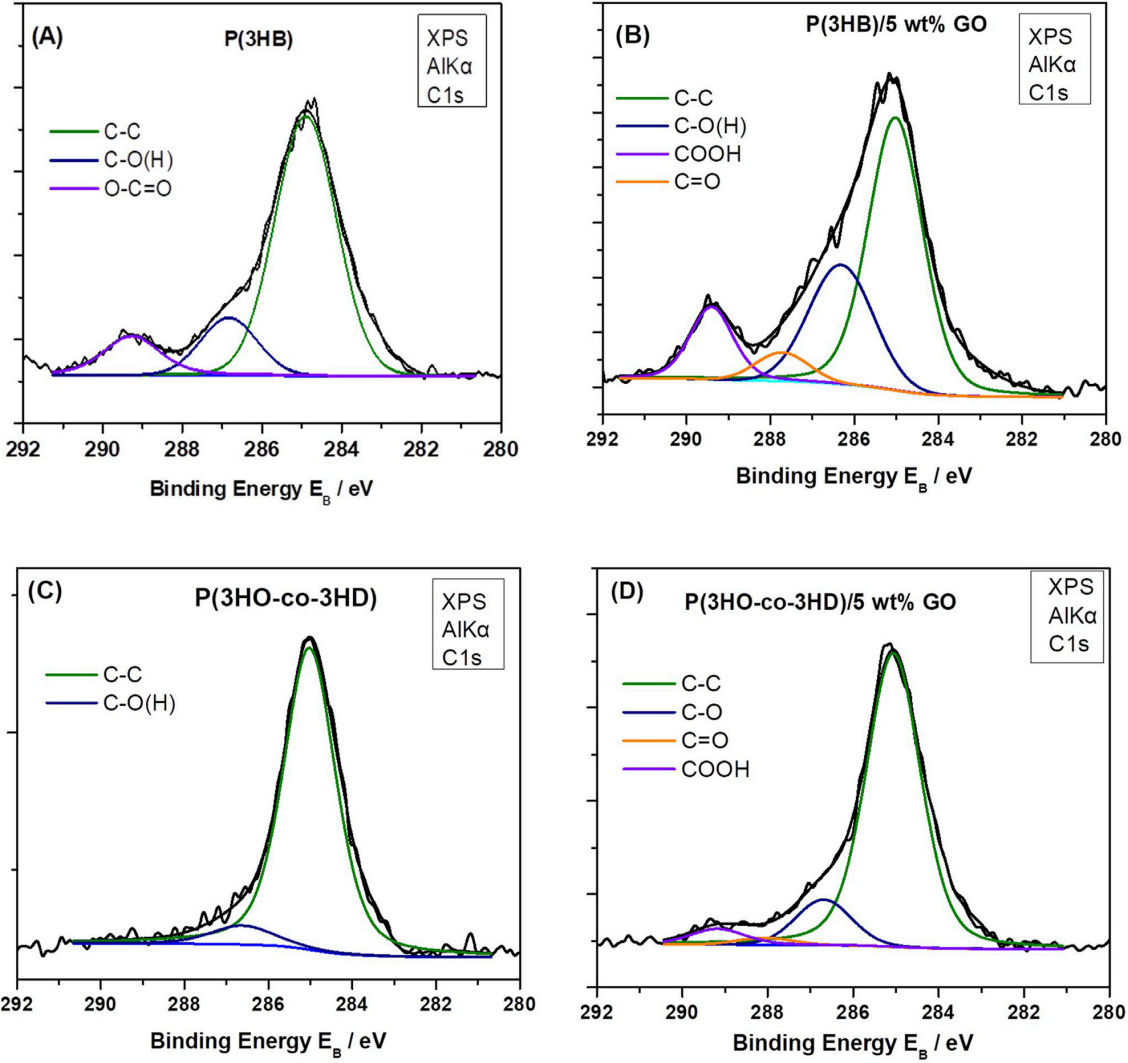
Deconvoluted C 1s XPS spectra of samples: **(A)** P(3HB) neat, **(B)** P(3HB)/5 wt% GO, **(C)**, neat P(3HO-*co*-3HD), and **(D)** P(3HO-*co*-3HD)/5 wt% GO.

The graphene oxide has been previously investigated by the FORTH group (Sygellou et al., 2015), and the C 1s XPS spectrum consisted of five components attributed to carbon–carbon bonds in C–C sp2 and defective (C–C) sp3 configuration and to carbon–oxygen bonds, namely, hydroxyl/epoxides (C–OH and C–O–C), carbonyls (C=O), and carboxyls (O=C-OH). Furthermore, the relative atomic concentrations (at%) of carbon and oxygen in the graphene oxide film were quantified from the C 1s and O 1s peak areas, revealing an oxygen concentration of approximately 35%. In the present study, an analogous analysis for the C 1s was performed. In the neat P(3HB) sample (Figure 5A), the C 1s consists of carbon in graphitic type (C–C), carbon oxygen single bonds, hydroxides or epoxides, and carboxyls (-COOH) as indicated from the P(3HB) chemical structure. In the P(3HB)/5 wt% GO composite (Figure 5B), the intensity of the aforementioned carbon–oxygen components (hydroxyls and carboxyls) increased considerably, accompanied by the appearance of a new component assigned to the carbonyl group (C=O), confirming the presence of GO in the composite. Similar results are depicted from the C 1s XPS peak of P(3HO-*co*-3HD) neat and P(3HO-*co*-3HD)/5 wt% GO composite (Figures 5C,D, respectively). In the neat material, the C 1s spectrum is mainly composed of the C–C component with a low-intensity C–O signal, whereas carbonyl and carboxyl peaks appeared in the composite, indicating the presence of GO. Finally, the relative atomic concentration (at%) of carbon and oxygen was calculated from the total peak area of the O 1s and C 1s peaks in each sample, divided by the appropriate sensitivity factors (based on Wagner’s collection and adjusted to the transmission characteristics of the analyzer EA10) (within experimental error ∼10%). The results are summarized in Table 1 along with the relative component concentration derived from the C 1s peak deconvolution.

**TABLE 1:** Relative atomic concentrations (at.%) of carbon and oxygen and percentages of epoxide–hydroxide (C–O–C, C–O(H)), carbonyl and carboxyl components in the raw and composites materials, as determined from XPS analysis (deconvolution of the C 1s XPS peak).

Sample	Atomic concentration		C 1s component concentration			
	% atomic O	% atomic C	% C–C	% C–O–C, C–O(H))	C=O	O=C–OH
P(3HB) neat	36	64	73	15	0	12
P(3HB) 5 wt% GO	36	64	56	26	5	12
P(3HO-*co*-3HD) neat	37	63	91	9	0	0
P(3HO-*co*-3HD)/5 wt% GO	40	60	82	12	1	4

As demonstrated in Table 1, an increase in the concentration of C 1s components related to oxygen-containing moieties, including hydroxyls/epoxides, carboxyls, and carbonyls, is evident in both the P(3HB) and P(3HO-*co*-3HD) composites with 5 wt% GO. This observation serves to confirm the presence of graphene oxide. However, it is notable that the total oxygen concentration increases only in the P(3HO-*co*-3HD)/5 wt% GO composite, while it remains constant in the P(3HB)/5 wt% GO composite, equivalent to the neat material. One hypothesis to explain this is that, in the neat P(3HB) polymer, the oxygen atoms are replaced by the oxygen moieties from the GO content in the composite.

#### 3.1.4 Raman spectroscopic characterization of the PHA and PHA/GO composite films

The structural fingerprint of the neat P(3HB) and P(3HO-*co*-3HD) matrix and their respective composites was investigated using Raman spectroscopy. The vibrational spectra of GO include the G band (1,318 cm^-1^) and D band (1,588 cm^-1^), which are assigned to the in-plane vibrations of bonded carbon atoms and the out-of-plane vibrations (disorder) associated with structural defects such as vacancies, grain boundaries, and amorphous carbon species, respectively (Qin et al., 2016; Strankowski et al., 2016).

The characteristic G and D peaks of the GO observed for the GO powder at 1,318 and 1,588 cm^-1^, respectively, confirm the successful synthesis of GO, and the ratio of these peaks (I_D_/I_G_) expresses the extent of disorder of the graphite materials. GO produced in this study was characterized as D-band dominant over the G-band, confirming the amorphization of graphite due to chemical modification.

**FIGURE 6:**
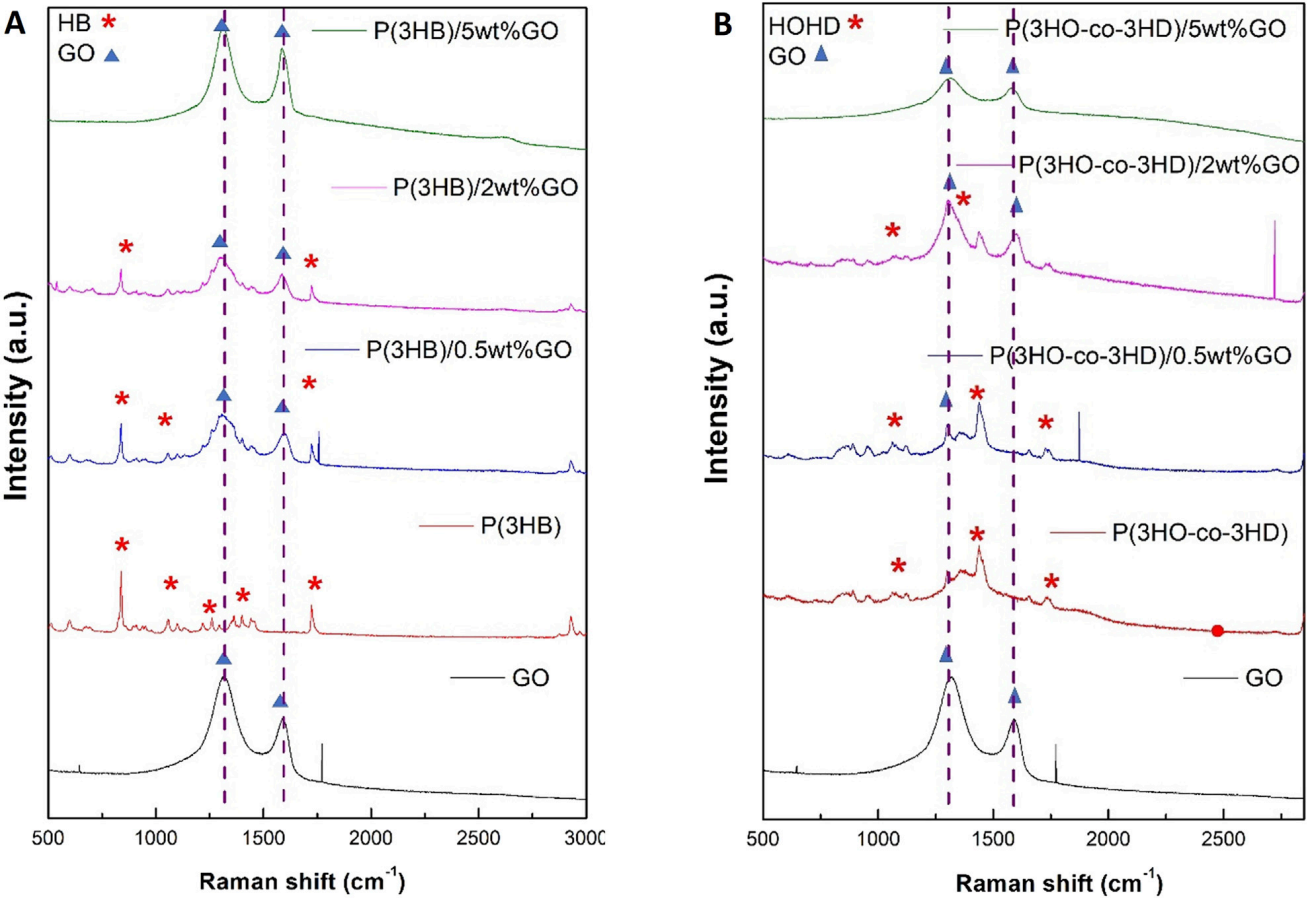
Raman spectra of **(A)** GO, P(3HB), and its composites containing 0.5 wt%, 2 wt%, and 5 wt% GO and **(B)** P(3HO-*co*-3HD) and its composites containing 0.5 wt%, 2 wt%, and 5 wt% GO.

Figure 6A shows the Raman spectra recorded for pure GO (in powder form) and the surfaces of the film samples (Figure 2) of P(3HB) and the corresponding P(3HB)/GO composites containing 0.5, 2, and 5 wt% GO. The Raman spectrum of pure GO shows, in addition to two sharp peaks at 646 and 1,771 cm^-1^, two broader bands of higher intensity at 1,315 and 1,597 cm^-1^. In the Raman spectrum of the P(3HB) film, 19 signals are visible in the wavenumber range from 500 to 3,000 cm^-1^ The peak characteristics of the repeat unit of this polymer are marked with asterisks in Figure 6A. The values of the intensity ratio (I_D_/I_G_) of the P(3HB)/GO composites were close to those of pristine GO, as shown in Table 2.

**TABLE 2:** D and G band position and ratio of I_D_/I_G_ for different composites containing GO.

Sample	D peak (cm^-1^)	G peak (cm^-1^)	I_D_/I_G_ ratio
GO	1,318	1,588	1.2
P(3HB)	-	-	-
P(3HB)/0.5 wt% GO	1,315	1,597	1.2
P(3HB)/2 wt% GO	1,300	1,589	1.2
P(3HB)/5 wt% GO	1,311	1,585	1.2
P(3HO-*co*-3HD)	-	-	-
P(3HO-*co*-3HD)/0.5 wt% GO	-	-	-
P(3HO-*co*-3HD)/2 wt% GO	1,306	1,603	1.2
P(3HO-*co*-3HD)/5 wt% GO	1,320	1,588	1.2

Similarly, for the P(3HO-*co*-3HD) composites, the asterisk on the Raman spectra is ascribed to the characteristic peaks of the co-polymer, where the detection of the G and D bands was feasible only for the composites containing 2 and 5 wt% GO (Figure 6B).

#### 3.1.5 X-ray diffraction analysis of the PHA and PHA/GO composite films

**FIGURE 7:**
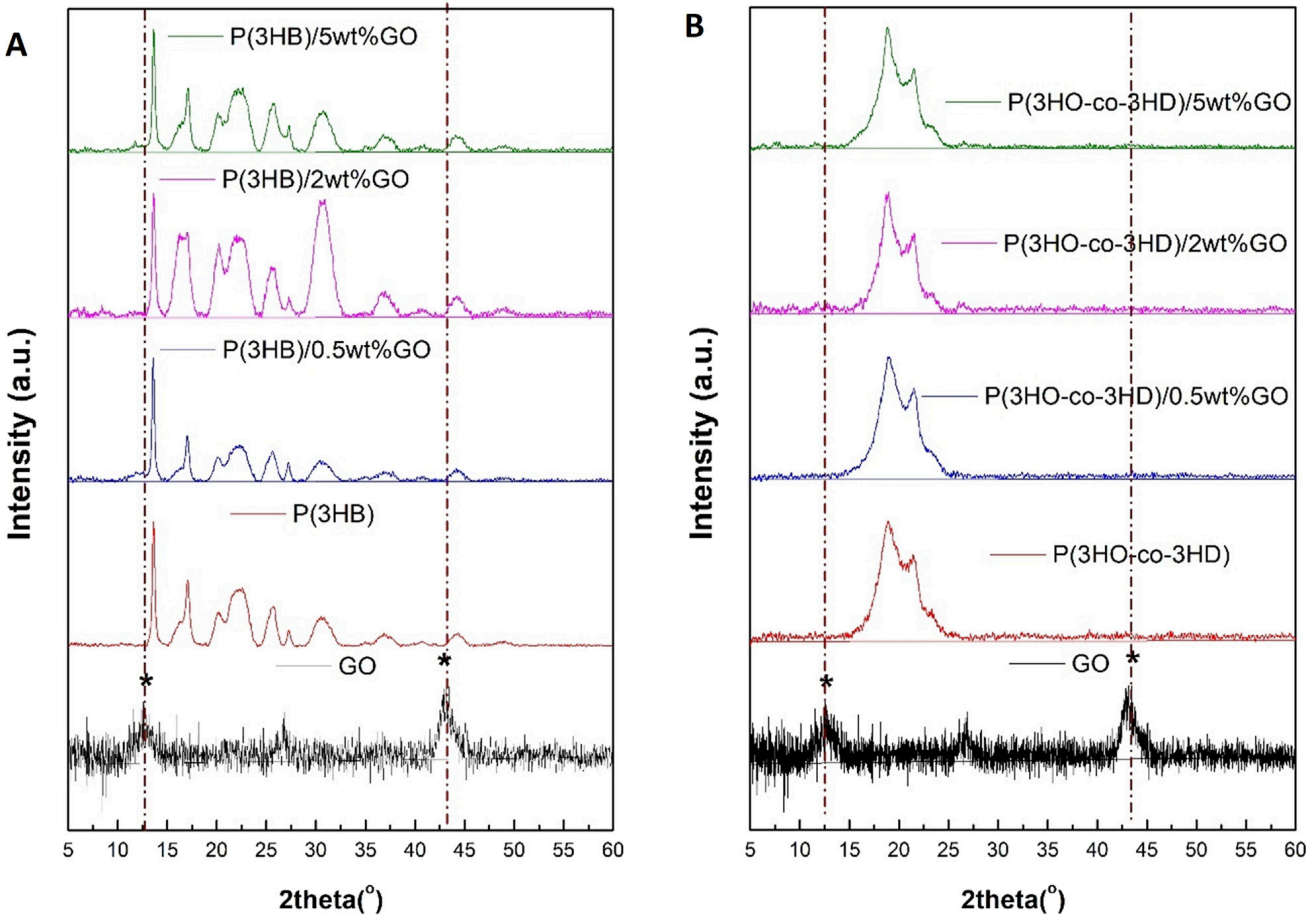
XRD diffractograms of **(A)** GO, P(3HB), and its composites containing 0.5 wt%, 2 wt%, and 5 wt% GO and **(B)** P(3HO-*co*-3HD) and its composites containing 0.5 wt%, 2 wt%, and 5 wt% GO.

X-ray diffraction studies have been carried out on the pristine GO, PHAs, and their respective composites, as shown in Figure 7.

The crystalline lattice of P(3HB) is reflected with the diffracted peaks shown in Figure 7A.

The XRD spectra revealed peaks located at 13.6°, 17°, 20°, 22°, 25°, 27°, and 44° for the neat P(3HB) film. The obtained peak positions for the homopolymer are in agreement with previously published studies (Mohapatra et al., 2017; Anbukarasu et al., 2015). The two characteristic peaks of the GO diffraction pattern are located at 2θ = 12° and 44°. The P(3HB) composites loaded with 0.5, 2, and 5 wt% GO revealed diffraction peaks assigned only to the P(3HB) matrix. The 2 and 5 wt% P(3HB)/GO composites exhibited broader characteristic peaks of the P(3HB). Similarly, in the case of the P(3HO-*co*-3HD) composites with the different ratios of GO loading (0.5, 2, and 5 wt%), no diffraction peaks corresponding to GO were detected (Figure 7B).

In addition to the surface structure analysis of the film surfaces made of the homopolymer P(3HB) and the copolymer P(3HO-*co*-3HD) and the corresponding PHA/GO composite films, the results of the DSC measurements are given below in order to obtain thermodynamic information on the material bulk structure.

#### 3.1.6 Differential scanning calorimetry analysis of the PHA and PHA/GO composite films

DSC was used to investigate the thermal properties of the bio-polyesters and their respective composites. The effects of the different GO fractions (0.5, 2, and 5 wt%) on the P(3HB) and P(3HO-*co*-3HD) structure of the respective PHA/GO composite films compared to the pure polymer films are characterized by means of the thermodynamic parameters of glass transition temperature (T_g_), the melting temperature (T_m_), and enthalpy of fusion (ΔH_m_) (Table 3).

**TABLE 3:** Thermal properties of P(3HB) and P(3HO-*co*-3HD) composites, which contain different amounts of graphene oxide.

Sample	T_g_ (^°^C)	T_m_ (^°^C)	ΔH _m_(J/g)
P(3HB)	−4	174	67.4
P(3HB)/0.5 wt% GO	−1.8	172.6	61.6
P(3HB)/2 wt% GO	0.5	172.3	81.5
P(3HB)/5 wt% GO	−0.1	172.7	71.9
P(3HO-*co*-3HD)	−45	54	1.1
P(3HO-*co*-3HD)/0.5 wt% GO	−49.3	61.3	0.3
P(3HO-*co*-3HD)/2 wt% GO	−51.3	60	1.3
P(3HO-*co*-3HD)/5 wt% GO	−48.6	59	1.7

T_m_ is the melting peak, T_g_ is the glass transition, and ΔH^n^_m_ is the enthalpy of fusion normalized to the mass fraction of the polymer.

The second heating cycle of the DSC thermograms was used to determine T_g_ and T_m_ values of the neat PHAs and their respective composites. The T_m_ value of P(3HB) is at 174 °C, while the P(3HB)/GO composites exhibited lower T_m_ values of 172.6 °C, 172.3 °C, and 172.7 °C and higher T_g_ values of −1.8, 0.5, and −0.5 for the 0.5, 2, and 5 wt% GO content, respectively. In the case of P(3HO-*co*-3HD), the T_m_ value is 54 °C, and the composites containing 0.5, 2, and 5 wt% GO had T_m_ values of 61.3 °C, 60 °C, and 59 °C. On the other hand, T_g_ values are −45 for the neat P(3HO-*co*-3HD) and −49.3, −51.3, and −48.6 for the respective composites of 0.5, 2, and 5 wt% GO.

The melting enthalpies of the specimens of the normalized values based on a mass fraction of the corresponding polymer are shown in Table 3.

The addition of 0.5 wt% of the antimicrobial filler into the polymer reduced ΔΗ_m_ to 61.6 compared with 67.4 J/g for pure P(3HB). On the other hand, further increases in GO content to 2 and 5 wt% GO resulted in melting enthalpies of 81.5 and 71.9 J/g, respectively, representing a substantial increase compared with pure P(3HB). At the highest GO loading (5 wt%), a lower ΔΗ_m_ value was observed than for the 2 wt% composite. The highest ΔΗ_m_ value was detected in the composite of P(3HB) containing 2 wt% GO.

Similarly to P(3HB), the P(3HO-*co*-3HD) composites containing the lowest GO fraction (0.5 wt.%) exhibited a decrease in the ΔΗ_m_ value of the composite by 68.6% compared to the neat polymer matrix. Further addition of the GO content (2 and 5 wt%) increased the melting enthalpy of the fabricated composites by 27% and 58%, respectively. The highest crystallinity for the P(3HO-*co*-3HD) composites was achieved with the addition of 5 wt% of GO.

The addition of the antibacterial agent has led to changes in crystallinity due to molecular structural changes, which can affect the mechanical properties of the samples. The results of the mechanical evaluation are shown below.

#### 3.1.7 Mechanical characterization of the PHA and PHA/GO composite films

The mechanical properties of the bio-polyesters and their respective composites were obtained in tensile testing. Film strips of the composites and their respective matrices were subjected to tensile testing in order to acquire the tensile strength, elongation at break, and Young’s modulus of the samples (Table 4).

**TABLE 4:** Mechanical properties of PHA/GO composites.

Sample	Tensile strength (σ) (MPa)	Elongation at break (ε_b_ %)	Young’s modulus (E) (MPa)
P(3HB)	20 ± 3.3	21.3 ± 1.8	776 ± 15.2
P(3HB)/0.5 wt% GO	15.7 ± 4.8	1.8 ± 0.3	968 ± 26.7
P(3HB)/2 wt% GO	18.4 ± 2.9	1.7 ± 0.3	1,055 ± 28
P(3HB)/5 wt% GO	13 ± 2.0	2.3 ± 0.1	839 ± 0.14.4
P(3HO-*co*-3HD)	9.8 ± 2.4	543 ± 69	7 ± 2.3
P(3HO-*co*-3HD)/0.5 wt% GO	7 ± 1.2	370 ± 42	9.1 ± 1
P(3HO-*co*-3HD)/2 wt% GO	4.5 ± 0.3	332 ± 60	10.1 ± 1.5
P(3HO-*co*-3HD)/5 wt% GO	3.8 ± 0.6	287 ± 34	12 ± 2.3

The addition of GO into the polymer matrix of P(3HB) increased the Young’s modulus, which reached a maximum value of 1,055 MPa for the composite containing 2 wt% GO. Amongst the P(3HB) composites, the highest value of the tensile strength was also observed for the 2 wt% GO P(3HB) composite (18 MPa) (2 MPa less than the neat polymer). The composite with higher GO loading (5 wt%) exhibited decreased Young’s modulus and tensile strength values compared with the 2 wt% composite (29% and 20.5%, respectively).

In the case of P(3HO-*co*-3HD) composites, the addition of the GO particles resulted in a decrease in elongation at break and an increase in Young’s modulus values compared with the P(3HO-*co*-3HD) copolymer. The lower GO loading level (0.5 wt%) decreased the tensile strength by 28.5%; however, the Young’s modulus was increased by 30% compared to the neat copolymer. Furthermore, the addition of GO (2 and 5 wt%) resulted in tensile strengths of 4.5 and 3.8 MPa and Young’s modulus values of 10.1 and 12 MPa, respectively. These values indicate a proportional correlation between the GO content and Young’s modulus; the higher the loading of the GO into the P(3HO-co-3HD) matrix, the higher the Young’s modulus values. Conversely, the elongation at break decreased with an increase in the GO content within the polymer (Table 4).

### 3.2 Biological characterization

#### 3.2.1 *In vitro* antibacterial evaluation of graphene oxide composites

The evaluation of the antibacterial properties of the composites was conducted by performing a direct contact test (DCT) for a time period of 24 h, adapted from the ISO 22196 (Japanese test method JISZ 2801) standard. Two types of bacterial strains, Gram-positive *Staphylococcus aureus* ATCC^®^ 29213™ and Gram-negative *Escherichia coli* ATCC^®^ 25922™, were incubated in direct contact with the composites and their respective neat matrices. The test provides quantitative results of the antibacterial activity of the composites with respect to the neat polymers.

**FIGURE 8:**
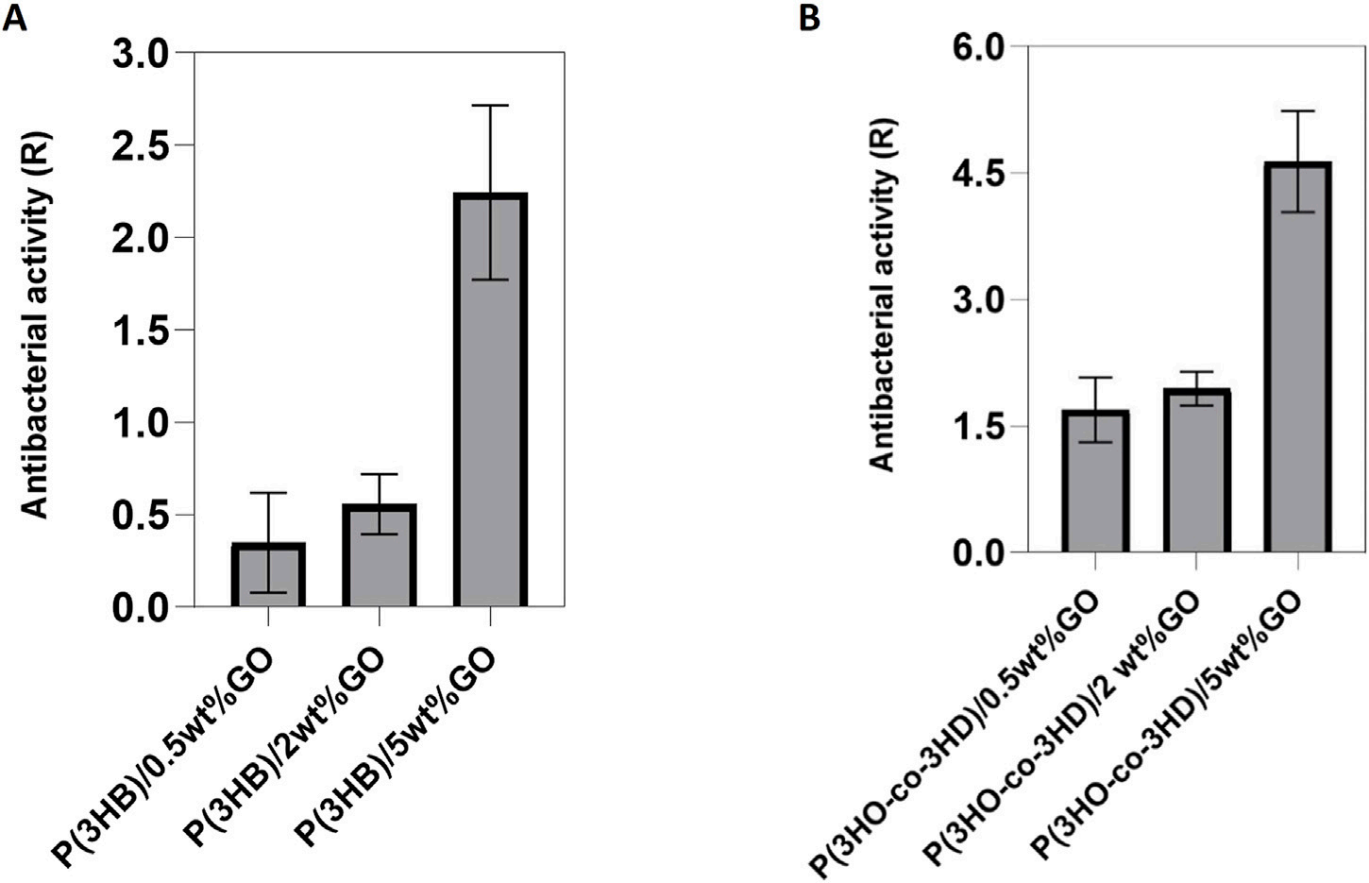
Antibacterial activity of **(A)** P(3HB) and **(B)** P(3HO-co-3HD) composites against Staphylococcus aureus ATCC^®^ 29213^™^. (Calculation of the resulting R values according to ISO22196/ JISZ 2801.

The results of the antibacterial activity of the composites against *S. aureus* ATCC^®^ 29213™ are shown in Figure 8. The obtained results indicated that an increase in the GO content resulted in a reduction in the bacterial cell growth on the surface of the composites (Figure 8A). Similar to the P(3HB) composites, the P(3HO-*co*-3HD) composites revealed the same trend; the increase in GO loading conferred higher antibacterial efficacy of the composites against sessile *Staphylococcus aureus* (ATCC^®^ 29213™).

**FIGURE 9:**
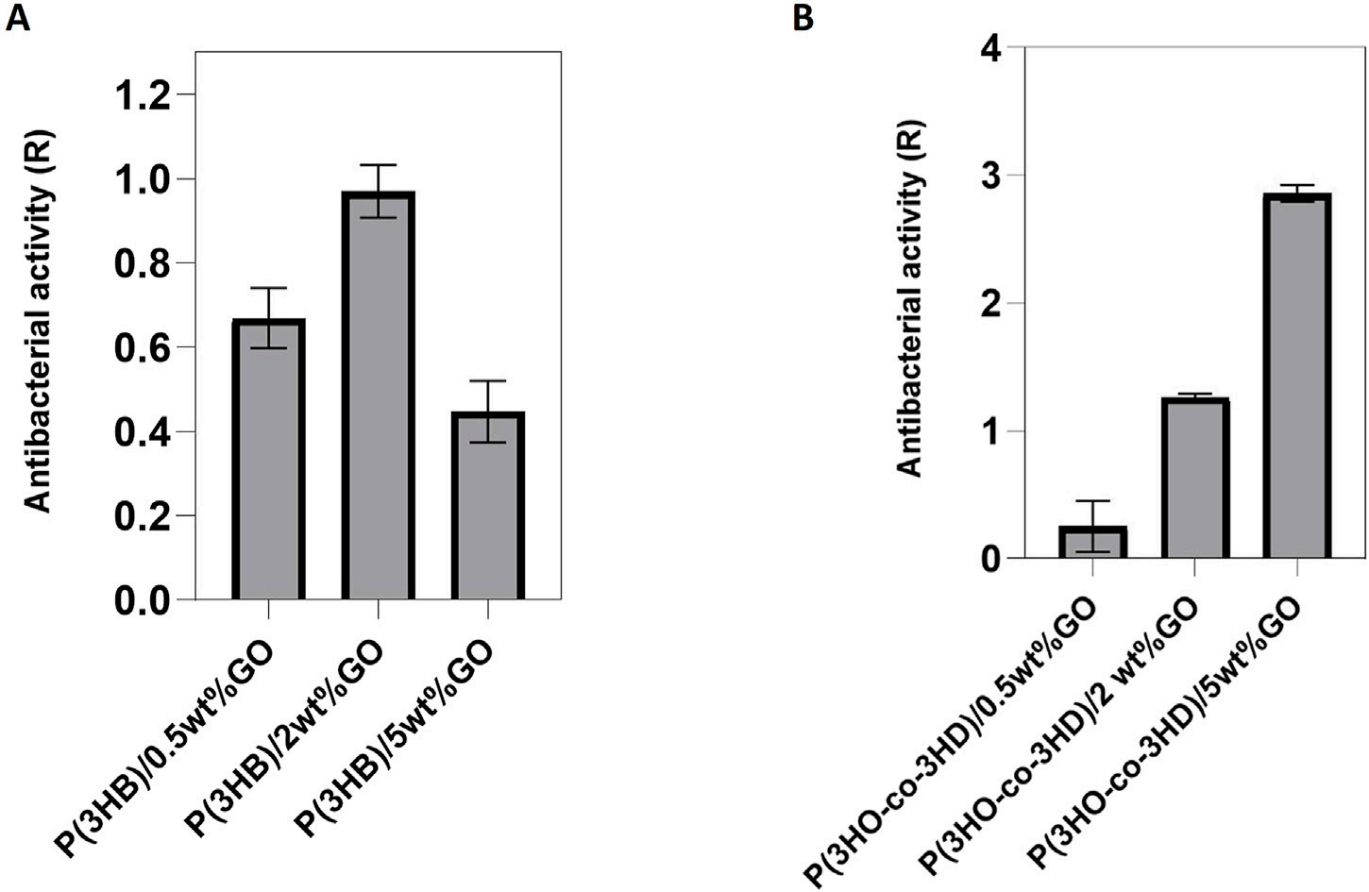
Antibacterial activity of **(A)** P(3HB) and **(B)** P(3HO-co-3HD) composites against Escherichia coli ATCC® 25922™. (Calculation of the resulting R values according to ISO22196/ JISZ 2801. Note that the R values were calculated to the growth controls at the respective time to avoid bias).

The same test was performed using Gram-negative *Escherichia coli* ATCC^®^ 25922™. After 24 h of incubation with the bacterial cells, the antibacterial activity of the P(3HB) composites was found to be higher for the composite with 2 wt% GO content (Figure 9A). However, in the case of the P(3HO-*co*-3HD) composites, a concentration-dependent correlation in antibacterial activity was observed. The higher the amount of GO in the polymer matrix, the higher the antibacterial activity (Figure 9B). The growth reduction for both the Gram-positive and Gram-negative bacterial cells upon their incubation with the PHA composites indicated that the resulted antibacterial activity conferred with the GO addition.

#### 3.2.2 *In vitro* direct cytotoxicity evaluation of PHA and composites

The cytotoxicity of the PHA/GO composites was assessed with direct *in vitro* seeding with respect to two different cell types, L929 murine fibroblasts and NG108-15 neuronal cells.

**FIGURE 10:**
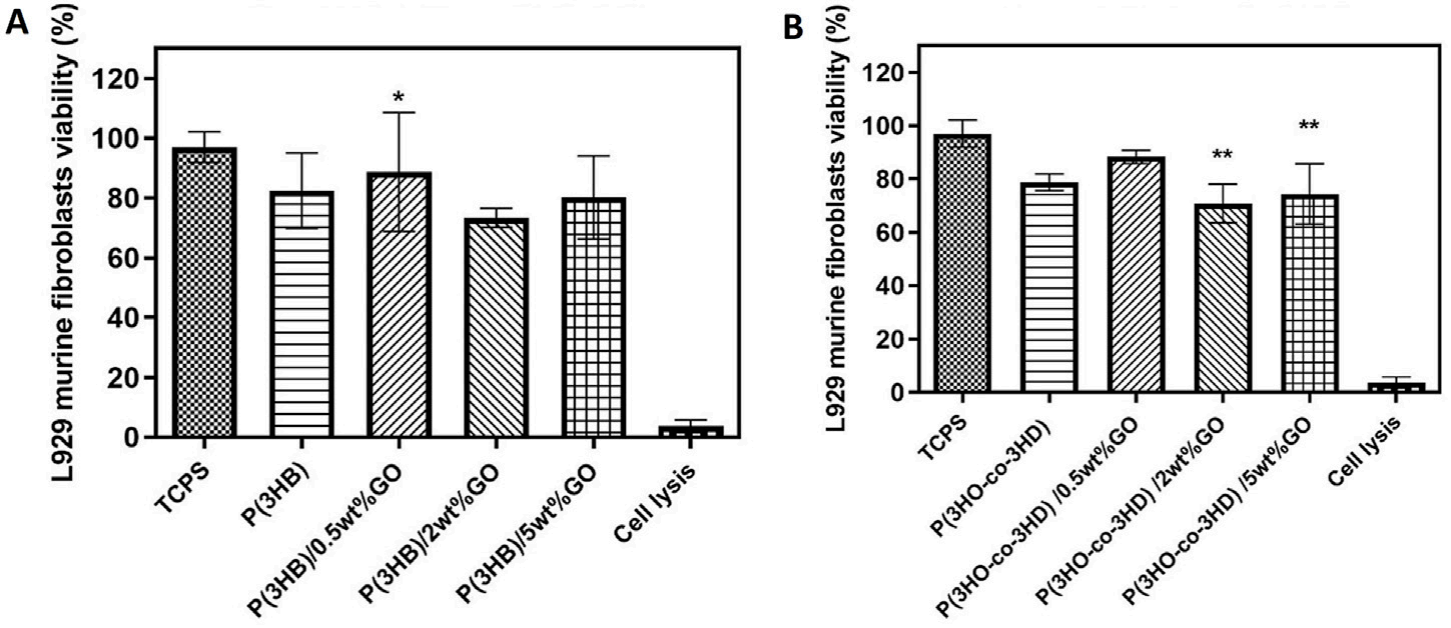
Direct cytocompatibility evaluation of **(A)** P(3HB) and **(B)** P(3HO-*co*-3HD) composites containing different loadings of graphene oxide with respect to L929 cells (mean ± SEM, n = 9 independent experiments). Statistical significance is represented as *p ≤ 0.05, **p ≤ 0.01, and ***p ≤ 0.001 for comparisons of cells on TCPS vs. GO composites.

First, the well-established L929 cell line was seeded on the surface of the neat biopolymers and their fabricated composites for a period of 24 h (Figure 10). The cytocompatibility was expressed as a percentage of cell viability relative to the positive control tissue culture polystyrene (TCPS).

The P(3HB) composites containing 0.5, 2, and 5 wt% showed cell viabilities of 89, 74, and 80%, respectively. Statistical analysis showed significant differences (*p ≤ 0.05) between the P(3HB)/0.5 wt% GO sample and the positive control (TCP) after 24 h of incubation (Figure 10A).

Statistical analysis of the composites of P(3HO-*co*-3HD) containing GO showed significant differences (**p ≤ 0.01) only between the TCPS and the samples of P(3HO-*co*-3HD)/2 wt% and P(3HO-*co*-3HD)/5 wt% for the first 24 h of incubation, as shown in Figure 10B. The addition of 0.5, 2, and 5 wt% GO to the polymer matrix resulted in a cell viability of 88%, 72%, and 75%, respectively.

**FIGURE 11:**
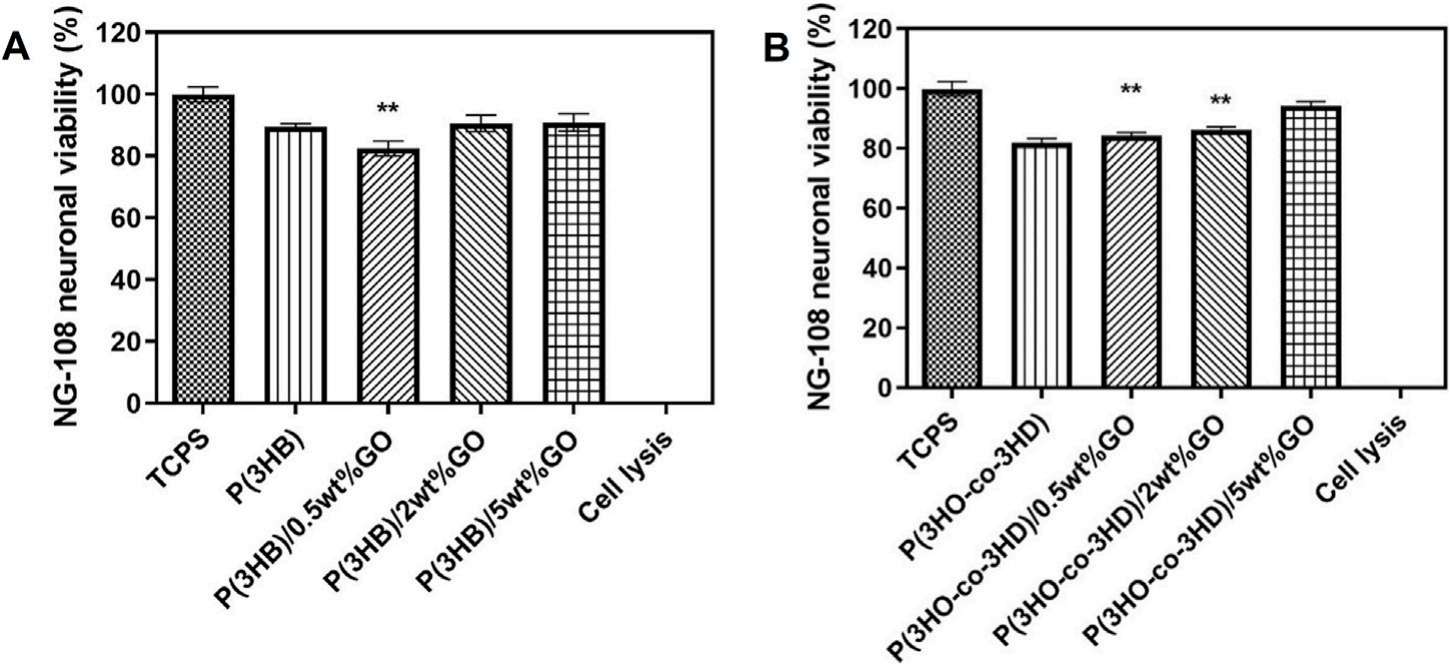
Direct cytocompatibility evaluation of **(A)** P(3HB) and **(B)** P(3HO-*co*-3HD) composites containing different loadings of graphene oxide with respect to NG108-15 cells (mean ± SEM, n = 9 independent experiments). Statistical significance is represented as *p ≤ 0.05, **p ≤ 0.01, and ***p ≤ 0.001 for cells on TCPS vs. PHA/GO composites.

To further investigate the cytocompatibility behavior of the fabricated composites, the samples were tested with respect to the NG108-15 cell line. The direct contact test was performed for a time period of 24 h. The assessment of cytocompatibility was expressed as cell viability percentage relative to the positive control TCPS (Figure 11).

After the first 24 h, the P(3HB) composites containing 2 and 5 wt% GO exhibited neural cell viability of 90%. Statistical analysis showed that there were significant differences (**p ≤ 0.01) between the P(3HB)/0.5 wt% composite and TCPS. The P(3HO-*co*-3HD) composites (0.5, 2, and 5 wt%) showed cell viability values of 85%, 86%, and 90%, respectively. Statistical analysis showed significant differences (**p ≤ 0.01) between the TCPS and 0.5 and 2 wt% composites.

**FIGURE 12:**
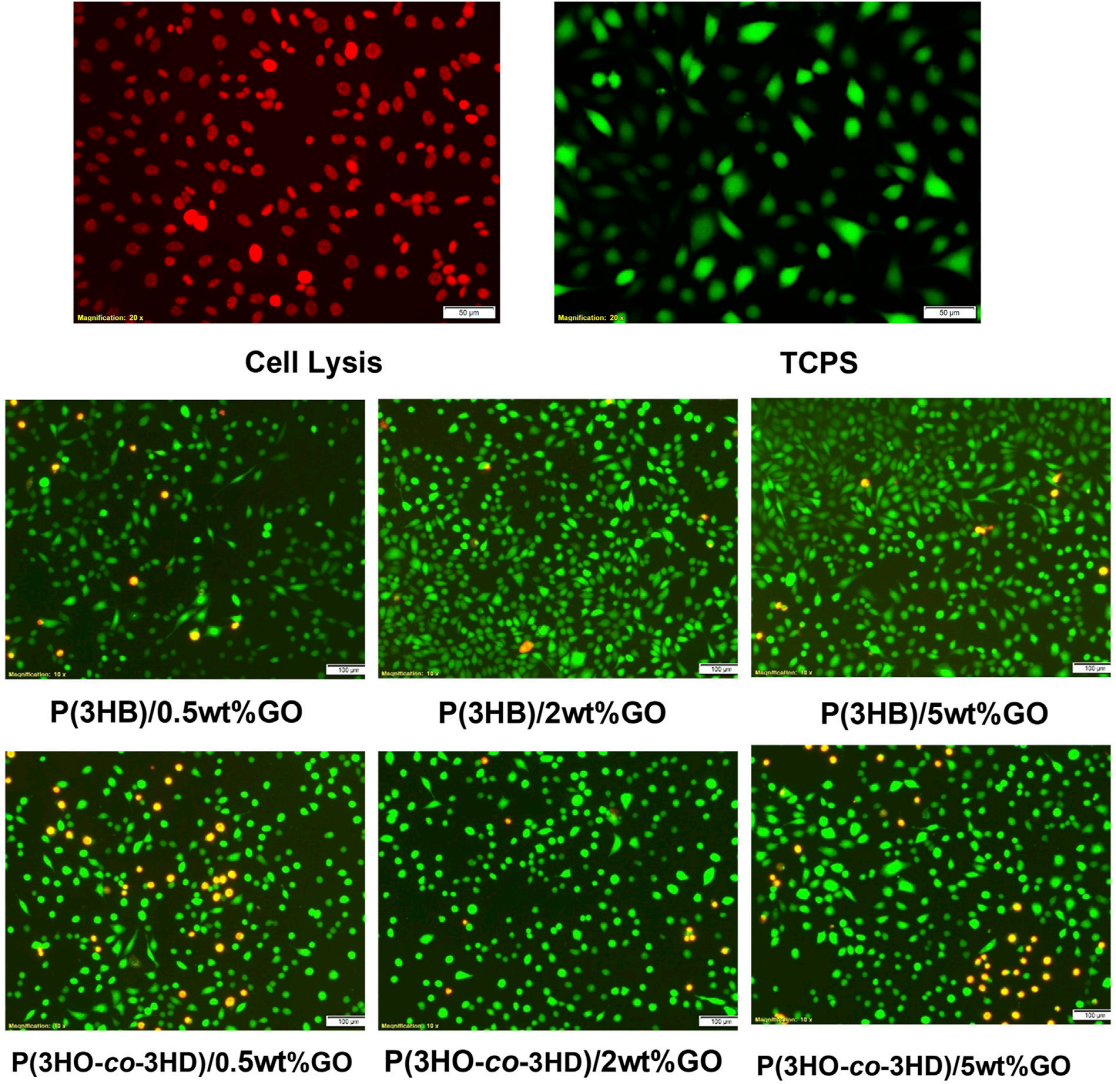
Fluorescence micrographs of NG108-15 neuronal cells stained with ethidium homodimer-III (red) and calcein AM (green) after 24 h in culture on the respective samples.

The fluorescent micrographs showed the presence of live cells (green) on the different PHA/GO composites. Additionally, the micrographs (Figure 12) revealed similar cell morphology on TCPS and PHA/GO composites.

## 4 Discussion

The modern way of living is accompanied by highly mobile populations across countries, which in turn aggravate the global antimicrobial resistance (AMR) problem. Despite technological advances in regenerative medicine, the proliferation and colonization of bacteria on implants remain a major cause of rejection and can result in increased post-surgery mortality. The multidisciplinary era of modern tissue engineering requires exploration of alternative approaches to prevent bacterial colonization and, consequently, biofilm formation on sensitive surfaces such as medical implants. Thus, there is an emerging need to develop implants that inhibit bacterial adhesion while simultaneously allowing eukaryotic cells to proliferate.

In this study, eco-friendly, non-toxic antibacterial materials were developed using a polymer matrix containing two different types of PHAs, scl and mcl PHAs, with antibacterial activity imparted by the addition of the inorganic compound, GO. Only a few studies have investigated the antibacterial properties and, at the same time, the cytotoxicity of PHA/GO composites on mammalian cells. Several mechanisms have been suggested in regard to the antibacterial actions of GO, including oxidative stress by reactive oxygen species (ROS) (Zhang N. et al., 2016; Panda et al., 2018; Wu et al., 2018), “sharp” edges of GO platelets, which cause damage to cell membranes, leading to leakage and death (Nanda et al., 2015; Ervina et al., 2016; Ji et al., 2016; Gao et al., 2017; Liu et al., 2018), and electron transfer interaction from microbial membranes to GO (Li et al., 2014; Ji et al., 2016). At the same time, there is a contradiction in the literature, as in some studies, the antibacterial activity of GO has not been observed (Bianco, 2013).

PHAs, the bio-polyesters with proven biocompatibility and biodegradability, were produced via bacterial fermentation (Mukherjee and Koller, 2023; Koller, 2018). With the aim of developing materials for tissue engineering with a wide range of mechanical properties and intrinsic antimicrobial activity, composites of P(3HB) and P(3HO-*co*-3HD) were prepared by introducing GO as a particulate filler at loading levels of 0.5, 2, and 5 wt%. The composites were produced by the solvent casting method, with the samples being produced as films with a thickness of approximately 200 µm. The crystallinity of GO used to make the composites drastically degraded, as shown by Raman spectroscopy with a D to G ratio of 1.2. Furthermore, intensities of peaks in GO XRD diffractograms decreased, indicating preservation of only a small fraction of ordered structures. For this ordered structure, the interlayer distance between GO platelets significantly increased.

Both visual inspection and SEM imaging demonstrated aggregation of GO particles within the polymer matrix. It needs to be noted that the solvent casting technique used in sample preparation is not in favor of GO platelet delamination and dispersion in the polymer matrix. Chloroform, one of the best solvents for PHAs, disperses GO only sparingly (Konios et al., 2014). Despite this poor GO dispersibility, its aggregates were uniformly distributed in the composites.

The SEM micrographs revealed the differences in the topography between the P(3HB) and P(3HO-*co*-3HD) films. The mcl-PHA samples exhibited a smoother surface than the scl-PHA samples, which showed an uneven morphology. The differences of the neat films most probably originated from the different degrees of crystallinity and flexibility of polymer chains between the scl and mcl-PHA. Due to a higher degree of crystallinity and faster crystallization of P(3HB) (Kai et al., 2003; Anbukarasu et al., 2015; Li et al., 2016), it solidifies in patches, resulting in uneven surfaces of the scl-PHA films. The surface morphology of the composites was analyzed following the addition of GO and compared with that of the neat polymers. The addition of different amounts of GO resulted in different effects on the polymer matrix of neat P(3HB) and P(3HO-*co*-3HD) films. The P(3HO-*co*-3HD) composites showed the least affected topography, whereas the P(3HB) composites exhibited various-sized pin-holes that were visible even with the lowest GO (0.5 wt%) loading. Analysis of the surface morphology evidenced differences between the P(3HB)-based and P(3HO-*co*-3HD-*co*-3HDD)-based samples. Mcl-PHA films obtained by solvent evaporation are usually characterized by a smooth surface, while scl-PHA films usually show a rougher surface with protrusions and pores (Rai et al., 2008; Anbukarasu et al., 2015; Kai et al., 2003; Li et al., 2016).

Surface morphology, along with molecular composition, defines the wettability of the surface, which is very often considered in the evaluation of surface hydrophobicity. The hydrophobicity/hydrophilicity of the samples was determined based on the contact angle: surfaces with a contact angle greater than 90° were considered hydrophobic (water repellent), whereas surfaces with a contact angle less than 90° were considered hydrophilic (Förch et al., 2009; Peschel et al., 2008; Basnett (2014). The water contact angle for the neat P(3HO-*co*-3HD) sample was higher than for P(3HB). This indicates that mcl-PHA is more hydrophobic, and this feature is ascribed to the long aliphatic chain of P(3HO-*co*-3HD), which demonstrates its hydrophobic feature. For both polyesters, incorporating hydrophilic GO caused a decrease in water contact angles. This indicates an exposure of the GO platelet at the composite surface. In the case of the P(3HB) composites, the larger decrease in water contact angle was observed for the composite with the 2 wt% filler content. An increase in the water contact angle with a further increase in GO content above 2 wt% is likely to be caused by surface roughening and GO aggregation. A similar trend was observed for P(3HO-*co*-3HD) composites.

XPS analysis revealed the enrichment of carbonaceous species on the surface of the composites. The deconvolution of the C 1s and O 1s peaks allowed the calculation of the percentage of the average relative atomic concentration for the neat polymers and their composites containing the highest amount of the filler (5 wt% GO). An increase in the presence of carbonyl (C=O) and carboxyl (O-C=OH) groups, attributed to the incorporation of GO in the polymer matrix, was observed only in the P(3HB)/5 wt% composite.

The Raman spectra of the nanocomposites and their respective neat matrices of PHAs were used to investigate the presence of GO, revealing the characteristic peaks of D and G. The presence of the carbonaceous component is more pronounced in the P(3HB)/GO composite than in the P(3HO-*co*-3HD)/GO composite although the amount of GO was exactly the same in both nanocomposites (0.5 wt%). The P(3HB) composite containing 0.5 wt% of the filler seems to exhibit a stronger resonance Raman effect, indicating that the short aliphatic chains did not disrupt the hydrogen bonds and other non-covalent interactions of the polymer with the oxygen molecules of GO (Bhawal et al., 2016; Keyte et al., 2019). In addition, the close values for the I_D_/I_G_ ratio of the composites compared with the value of GO indicated the well-preserved structure of GO. The P(3HO-*co*-3HD) composites containing 2 and 5 wt% of GO revealed both the characteristic peaks of D and G bands with a broader shape compared with pristine GO, indicating the incorporation of GO into the polymer host, which forms a much more disordered and defective structure than pure GO (Strankowski et al., 2016). For the 2 wt% composite, the upshift of the G band at 1,603 cm^-1^ may result from an enhanced and more efficient dispersion of the GO in the polymer due to the presence of oxygen-containing groups that can form bonds with the ester groups of the polymer and, hence, reduce the GO–GO interactions The intensity ratio (I_D_/I_G_) of the 2 wt% composite was higher than that of pristine GO, indicating a higher degree of defects in the composite material.

The XRD patterns revealed the solid-state structural properties of the polymers and their respective composites. The XRD peaks of the crystalline P(3HB) are indexed to the orthorhombic crystal planes (020), (110), (021), (111), (121), (040), and (222) of the P(3HB) film. Located at 2θ = 12° and 44° are the two characteristic peaks of GO, which are assigned to (001) and (100) planes with d-spacings of 7.3 Å and 2.1 Å respectively, confirming the successful synthesis of GO (Johra et al., 2014; Ameer and Gul, 2016; Ain et al., 2019). A well-formed network of the GO with polymer matrices of P(3HB) and P(3HO-*co*-3HD) is indicated by the absence of the characteristic XRD peak of GO located at 2θ = 12°, even for composites containing 5 wt.% GO. Moreover, the shifts of the XRD characteristic peaks of the PHA indicated a change in the crystallinity of the final composites compared with the respective neat matrices. In the case of the P(3HB)/GO composites, a more disordered structure was revealed since the characteristic peaks of the polymer matrix decreased in intensity compared with the respective PHA matrix. With the higher filler content of 5 wt%, a higher peak intensity was observed than the other composites, implying stacking of the graphene oxide layers within the P(3HB) matrix. In the 0.5 and 2 wt% composites of the same biopolymer (P(3HB)), the presence of low-intensity peaks indicates a more amorphous structure and the presence of defects on the final composites (Larsson et al., 2017). Similar findings have been reported in the literature when high molecular weight polyurethanes and ethylene methyl acrylate hybrid nanocomposites were developed with the addition of GO (Bhawal et al., 2016; Strankowski et al., 2016; Larsson et al., 2017).

The DSC analysis revealed the changes in the thermal properties and the crystallinity of the final composites upon the addition of different ratios of GO. The obtained composites showed insignificant changes in their thermal values (T_g_ and T_m_) compared to their respective neat matrices, indicating that compounding with GO has little effect on the thermal properties of the polymer matrices. There are no reported values in the literature for the heat of fusion for 100% crystalline P(3HO-*co*-3HD). Hence, the degree of crystallinity for P(3HO-*co*-3HD) could not be evaluated from the DSC results. Nevertheless, the degree of crystallinity is proportional to the enthalpy of fusion; thus, the change in crystallinity from the GO addition to the polymer matrices was considered in terms of the enthalpy of fusion (ΔΗ_f_) or the melting enthalpy (ΔΗ_m_), determined from the area under the melting endotherm. The higher values of ΔΗ_m_ indicate higher degrees of crystallinity of the samples. The addition of 2 and 5 wt% into the polymer matrix of P(3HB) enhanced the crystallinity of the composites, indicating that the GO particles acted as nucleation sites. The lower crystallinity observed for the 5 wt% composite, compared with the 2 wt% sample, is attributed to the barrier effect of the GO particles obstructing the growth of the crystals (Larsson et al., 2017). Similarly, the P(3HO-*co*-3HD) composites with 2 and 5 wt% GO showed an increase in the crystallinity of the polymer matrix. This phenomenon might be attributed to the decrease in free volume in the amorphous phase of the co-polyester due to the presence of GO, suggesting affinity between the filler and the polymer matrix via hydrogen bonds.

At the same time, the addition of GO increased both the crystallinity and rigidity of the respective matrices. For both types of PHAs, the introduction of GO improved the strength of the materials. The hydrogen bonding interactions between the ester groups of the bio-polyesters and the hydroxyl groups of GO enhanced Young’s modulus but reduced the elasticity compared with the neat matrices. The reinforcing effect of the GO on the polymer matrix has also been reported in other studies (Abdala et al., 2013; Sridhar et al., 2013; R. Mahendran et al., 2016; Larsson et al., 2017; Smith et al., 2019; Díez-Pascual, 2021).

More specifically, the P(3HB) composites exhibited greater Young’s modulus values compared with the neat polymer. This indicates that GO acts as a reinforcing filler, promoting more entanglements between the filler and the polymer matrix of the composite and enabling load transfer to the interphase. The composite containing the highest amount of the GO (5 wt%) showed a lower Young’s modulus compared with the 2 wt% composite. Such behavior can be ascribed to the increase in the filler interaction, more likely the restacking of GO, which reduces the reinforcement of the composite structure. Therefore, this agglomeration limits the load transfer from the matrix to GO particles, causing cracks that are easy to propagate. These cracks are reported to be responsible for the reduction in the strength of the developed composites (Ervina et al., 2016). Moreover, at higher GO loading, the bulk structure might have more defects due to GO aggregation in the polymer matrix. These findings are in agreement with the literature. Barrett et al. also mentioned that the addition of GO particles promoted additional entanglements between the polymer chain and the particles, enhancing the stiffness of the polymer matrix compared to the neat PHAs (Barrett et al., 2014; Mahendran et al., 2016; Smith et al., 2019). The elongation at break values in Table 4 show a marked decrease with increasing GO content for both P(3HB) and P(3HO-*co*-3HD) composites. This reduction in ductility can be attributed to the inherent stiffness of GO nanosheets, which act as rigid fillers that restrict polymer chain mobility. As the GO content increases, strong interfacial interactions between GO’s oxygen-containing functional groups and the polymer chains limit plastic deformation, leading to premature fracture under tensile load. Additionally, at higher loadings, GO aggregation can introduce stress concentration sites that act as crack initiation points, further reducing the material’s capacity to elongate before breaking. These effects explain the inverse relationship between GO content and elongation at break observed in this study. However, the final mechanical properties can further be optimized/tailored according to the required application, improving/weakening the interface between the polymeric matrix and the filler (Misra et al., 2006; Rai et al., 2008). This can be achieved by improving composite processing compared with the solvent-cast method, for example, through melt processing.

The fabrication of the composites aimed at developing 2D structures with antibacterial efficacy against pathogens found in the human body and nosocomial environments. Thus, they could be used as scaffolds for the prevention and reduction of bacterial colonization. The 2D composites were investigated for their antibacterial efficacy using the DCT—ISO 22196/JISZ 2801 (established for measuring antibacterial activity on plastics). Both types of composites containing the different fractions of GO exhibited high antibacterial activity on contact with the bacterial strains. The composites of P(3HB) and P(3HO-*co*-3HD) showed antibacterial activity against both Gram-positive and Gram-negative bacteria. Antimicrobial activity was concentration-dependent, increasing with the increase in GO content in the case of the P(3HO-*co*-3HD) composite (Figure 9B). The observed enhancement in antibacterial activity at higher GO loadings can be attributed to several factors. The increase in GO concentration enhances the number of exposed nanosheets on the composite surface, thereby increasing the available active surface area for bacterial contact. GO’s sharp edges can physically disrupt bacterial membranes, while its abundant oxygen-containing functional groups can induce oxidative stress via ROS generation. These mechanisms act synergistically to damage bacterial cells, and the probability of such interactions increases as GO content increases. This dose-dependent behavior is consistent with previous findings on graphene-based materials against both *S. aureus* and *E. coli*.

These results are in agreement with those of other studies reporting antibacterial activity of GO against both Gram-positive and Gram-negative species (Akhavan and Ghaderi, 2010; Mejías Carpio et al., 2012; Perreault et al., 2015; Panda et al., 2018; Díez-Pascual, 2021).

The addition of the GO particles into the bio-polyesters resulted in the development of inherently antibacterial composites. A comparison of the antibacterial activity of the P(3HB) and P(3HO-*co*-3HD) composites indicated that the samples showed greater antibacterial activity against Gram-positive *S. aureus* (ATCC^®^ 29213™) than the Gram-negative *E. coli* (ATCC^®^ 25922™). Other studies have previously reported that GO is toxic to bacteria and Gram-positive species are more susceptible than Gram-negative species (Akhavan and Ghaderi, 2010; Perreault et al., 2013; Perreault et al., 2015; Díez-Pascual, 2021). Interestingly, the antibacterial activity results showed that P(3HB)/2 wt% GO exhibited the highest antibacterial effectiveness, with an R value approaching 1.0, whereas the P(3HB)/5 wt% GO showed a lower antibacterial effect than both 2 wt% and 0.5 wt% GO composites. This observation suggests that while the addition of GO enhances antibacterial activity, excessive GO loading (5 wt%) may lead to agglomeration, reducing the effective surface area and limiting the contact between GO and bacterial cells. Consequently, 2 wt% GO appears to be the optimal concentration, balancing dispersion and activity to achieve maximum antibacterial performance.

Díez-Pascual (2021) tested the antibacterial activity of the poly(3-hydroxybutyrate-*co*-3-hydroxyhexanoate) composites containing a range of GO loading (0.5, 1, 2, and 5 wt%). The fabricated solvent casted films were successfully tested for their antibacterial activity against Gram-positive (*S. aureus* ATCC^®^ 12600 and *Bacillus subtilis* ATCC^®^ 23857™) and Gram-negative (*E. coli*, ATCC^®^ 25922™ and *B. cepacia*, ATCC^®^ 25416™). The superior antibacterial activity of the composites toward the Gram-positive species was ascribed to the different bacterial membrane composition. Additionally, Akhavan and Ghaderi (2010) investigated the antibacterial effect of graphene nanosheets against both *E. coli* and *Staphylococcus aureus* bacteria and reported that *E. coli* was more resistant to GO than *S. aureus*. In another study, Gao et al. (2017) observed that when *S. aureus* and *E. coli* bacteria were exposed to the same concentration of GO (40 mg/L), the antibacterial inactivation of the *S. aureus* strain was completed in a shorter period (12 h) than the *E. coli* (168 h). The different toxicity is mainly attributed to the different form and structure of the cell membrane of the *S. aureus* (spherical in shape (840 nm)) and *E. coli* (rhabdoid in shape (approximately 1,230–3,538 nm)) strains. The different composition of the cell membrane can affect how GO interacts with and disrupts the membrane. This difference in damage was evaluated by comparing the RNA concentration obtained from the supernatant of the bacterial strain samples. It was shown that the RNA released from the *S. aureus* samples was greater than that of the *E. coli* samples (Gao et al., 2017). Other suggested chemical and physical mechanisms of the antimicrobial action of GO include electron transfer and oxidative stress resulting from the generation of ROS, membrane stress, and the wrapping/entrapment of bacteria, facilitated by physical contact. Bacterial respiration is essential for the growth and metabolism of bacterial cells based on electron transport to extracellular electron acceptors. When bacterial cells interact with GO, surface proteins exhibiting n-type semiconducting behavior facilitate the formation of a Schottky barrier. This leads to electron transfer from the cell membranes to the graphene materials, which are effective electron acceptors, driven by Fermi level alignment (Prasad et al., 2017; Díez-Pascual, 2021).

The difference in surface electron states of the Gram-positive and Gram-negative bacterial membranes might be a reason for the differences in the antibacterial efficacy of the graphene materials against the two categories of bacterial species.

The ROS species generated from GO as hydroxyl radicals can attack the carbonyl groups of the peptide linkages of the bacterial cell wall and damage the cellular components. More specifically, they are responsible for the oxidation of the cellular components of the bacterial strains, causing peroxidation of lipids, enzyme inhibition, oxidation of proteins, and damage to nucleic acids, which results in microbial death (Prasad et al., 2017; Sharma et al., 2018; Díez-Pascual, 2021; Seifi and Ali, 2021). The physical mechanisms include the wrapping/entrapment of bacterial cells, lipid extraction, and the direct contact of sharp GO edges with bacterial membranes, inducing membrane stress and leading to membrane disruption and subsequent leakage of intracellular material (Akhavan and Ghaderi, 2010; Prasad et al., 2017; Díez-Pascual, 2021; Seifi and Ali, 2021). Hence, the presence of the outer membrane in the Gram-negative *E. coli* could act as protection against GO damage, while Gram-positive *S. aureus* lacks an outer membrane, thereby increasing the chances of cell death.

Nevertheless, further studies should be conducted to standardize the media, reagents, and methods, along with ensuring the process of GO origin, size, and other vital physicochemical features prior to antibacterial testing of such materials, so a more comparative correlation would be of most use for the future. Additionally, systematic studies are required for graphene and its derivatives before their incorporation with other constituents.

*In vitro* direct cell culture experiments were carried out in the presence of the PHA-based composites using L929 murine fibroblasts and NG-10815 neuronal cells. The results showed that the fabricated composites of both polymer types exhibited cytocompatibility toward both cell lines. The quantitative assessment of the samples over period of 24 h revealed that the fabricated composites favor the attachment and the proliferation of L929 murine fibroblasts and NG108-15 neuronal cells.

Chitosan composites loaded with different ratios of GO showed an enhanced bioactivity of the scaffold material when tested with respect to VERO from the African green monkey cell line and murine preosteoblasts MC3T3-E1cells. This behavior appeared to be proportional to the GO concentration (Dinescu et al., 2014). In another study, composites of polyvinyl-N-carbazole with GO exhibited a tolerable cytotoxic effect toward NIH 3T3 fibroblast cells (Mejías Carpio et al., 2012). Akhavan et al. (2014) conducted *in vitro* viability tests on ginseng-reduced graphene oxide sheets using human neural stem cells (hNSCs). The study showed that the ginseng-reduced graphene oxide showed better proliferation of the hNSCs compared to the rest of the samples. Hybrid nanofibrous scaffolds of PCL-GO were fabricated to guide rat neural stem cell (NSC) differentiation into oligodendrocytes. The authors reported that the selective differentiation of NSCs to mature oligodendrocytes occurred in the absence of inductive factors in the culture medium (Shah et al., 2014). Moreover, proliferation and osteogenic differentiation of adult MSCs isolated from goat bone marrow aspirates were successfully observed after coating culture plates with GO (0.1 mg/mL) (Hoda et al., 2015). Additionally, aligned PLLA nanofibrous scaffolds coated with graphene oxide promoted Schwann cell (SC) growth by regulating cell orientation and induced the differentiation and neurite outgrowth of rat pheochromocytoma 12 (PC12) cells (Zhang K. et al., 2016). When scaffolds of 2D graphene films and nanostructured rGO microfibers were seeded with NSCs, it was shown that the rGO microfibers exhibited superior proliferation and differentiation of the cells compared with the tissue culture plate and 2D graphene films. Over a time period of 5 days, the rGO scaffolds were able to regulate the NSC differentiation into neurons, forming a dense neural network similar to a functional nerve graft (Guo et al., 2017). The ability of pristine graphene to promote cell adhesion and proliferation of monkey renal fibroblast (Cos-7), as well as on primary E18 rat hippocampal neurons, was reported by Rastogi et al. (2017). They found that graphene does not induce any cell stress since no alteration of the mitochondrial membrane potential (MMP), mitochondrial morphology, and autophagy levels was observed after 9 days of incubation.

Despite these promising findings and the favorable attachment and proliferation of cells reported in the literature, some studies have reported that graphene-based materials exert significant toxic effects on PC12 cells in a dose- and time-dependent manner (Kang et al., 2017).

Interestingly, the antibacterial trends were not identical for the two polymer systems. In the case of P(3HB), the 2 wt% GO composites showed the strongest antibacterial activity, while higher loading (5 wt%) seemed to reduce activity, most likely because of GO agglomeration that limited the effective contact with bacterial cells. In contrast, for the P(3HO-*co*-3HD) composites (Figure 9B), antibacterial activity kept increasing with higher GO content. This difference suggests that the polymer matrix itself plays an important role in how well GO is dispersed. In P(3HO-*co*-3HD), the GO appears to remain better distributed even at higher concentrations, allowing its antibacterial mechanisms to scale with loading. These results highlight that GO’s antibacterial effect is not only related to concentration, but it also depends strongly on the surrounding polymer environment, which is consistent with other studies reporting GO activity against both Gram-positive and Gram-negative species (Akhavan and Ghaderi, 2010; Mejías Carpio et al., 2012; Perreault et al., 2015; Panda et al., 2018; Díez-Pascual, 2021).

## 5 Conclusion

In this research, two types of bio-polyesters served as matrices for creating GO composites. The study investigated both the physicochemical and biological characterization of the resulting scaffolds, aiming to address the global issue of antibacterial resistance in biomedical applications. The introduction of varying GO ratios (0.5, 2, and 5 wt%) had distinct impacts on the physicochemical attributes of the two different types of bio-polyesters (P(3HB) and P(3HO-*co*-3HD)). Physicochemical analyses confirmed uniform GO incorporation and surface morphological changes, while mechanical testing demonstrated that the addition of 2 wt% GO to P(3HB) increased the Young’s modulus from 776 ± 15 MPa to 1,055 ± 28 MPa, indicating enhanced stiffness. Both types of composites demonstrated antibacterial effectiveness against both Gram-positive and Gram-negative bacterial strains. According to ISO 22196 testing, GO demonstrated a concentration-dependent activity against *S. aureus* and *E. coli*, with P(3HB)/2 wt% GO achieving the highest antibacterial efficacy. Cytocompatibility testing using L929 fibroblasts and NG108-15 neuronal cells in direct contact with the specimens showed no adverse effects on cell viability, even in specimens with the highest concentration of GO. The processability of these composites offers a versatile platform for scaffold formulation, allowing adjustment of mechanical and thermal properties by modifying the GO content to meet specific application requirements. Furthermore, the developed drug-free composites exhibited targeted toxicity against bacterial cells without displaying any harm to mammalian cells. Overall, this study underscores the potential of these innovative antibacterial, biodegradable, highly biocompatible, and customizable composites for various medical applications. Future research will focus on fabricating 3D PHA/GO scaffolds and exploring their physicochemical and biological properties for potential use in nerve tissue engineering. In addition, *in vivo* evaluations, long-term antibacterial studies, and optimization of GO dispersion will be included to maximize efficacy and safety in clinical applications.

## Data Availability

The raw data supporting the conclusions of this article will be made available by the authors, without undue reservation.
